# Delivering the Promise of Gene Therapy with Nanomedicines in Treating Central Nervous System Diseases

**DOI:** 10.1002/advs.202201740

**Published:** 2022-07-18

**Authors:** Meihua Luo, Leo Kit Cheung Lee, Bo Peng, Chung Hang Jonathan Choi, Wing Yin Tong, Nicolas H. Voelcker

**Affiliations:** ^1^ Monash Institute of Pharmaceutics Science Monash University Parkville Campus, 381 Royal Parade Parkville VIC 3052 Australia; ^2^ Australian Institute for Bioengineering and Nanotechnology the University of Queensland St Lucia QLD 4072 Australia; ^3^ Department of Biomedical Engineering The Chinese University of Hong Kong Shatin New Territories Hong Kong; ^4^ Frontiers Science Center for Flexible Electronics Xi'an Institute of Flexible Electronics (IFE) and Xi'an Institute of Biomedical materials & Engineering Northwestern Polytechnical University Xi'an 710072 China; ^5^ Commonwealth Scientific and Industrial Research Organization (CSIRO) Clayton VIC 3168 Australia; ^6^ Melbourne Centre for Nanofabrication Victorian Node of the Australian National Fabrication Facility 151 Wellington Road Clayton VIC 3168 Australia; ^7^ Materials Science and Engineering Monash University 14 Alliance Lane Clayton VIC 3800 Australia

**Keywords:** bio‐nanotechnology, blood‐brain barrier, central nervous system diseases, gene therapy, nanomedicine

## Abstract

Central Nervous System (CNS) diseases, such as Alzheimer's diseases (AD), Parkinson's Diseases (PD), brain tumors, Huntington's disease (HD), and stroke, still remain difficult to treat by the conventional molecular drugs. In recent years, various gene therapies have come into the spotlight as versatile therapeutics providing the potential to prevent and treat these diseases. Despite the significant progress that has undoubtedly been achieved in terms of the design and modification of genetic modulators with desired potency and minimized unwanted immune responses, the efficient and safe in vivo delivery of gene therapies still poses major translational challenges. Various non‐viral nanomedicines have been recently explored to circumvent this limitation. In this review, an overview of gene therapies for CNS diseases is provided and describes recent advances in the development of nanomedicines, including their unique characteristics, chemical modifications, bioconjugations, and the specific applications that those nanomedicines are harnessed to deliver gene therapies.

## Graphical abstract

The review provides an up‐to‐date overview of various cutting‐edge gene therapies for central nervous system (CNS) diseases and describes most recent advances in the development of nanomedicines, outlining their characteristics, potential opportunities, and challenges in clinical applications. The systematic review of nanomedicine approaches provides cross‐disciplinary insights to enable the realization of advanced gene‐therapies for the treatment of CNS diseases.

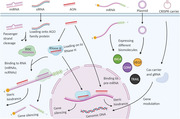

## 1 Introduction

Central nervous system (CNS) diseases, including Alzheimer's disease (AD), Parkinson's disease (PD), stroke, Huntington's disease (HD), brain tumors, and multiple sclerosis, are the leading cause of disability and the second leading cause of mortality worldwide.^[^ ^1^ ^]^ Globally, the population affected by CNS diseases has substantially increased from 1990 to 2015. In 2016, CNS diseases resulted in 9.0 million deaths, contributing 16.5% of total deaths from all causes over the world.^[^ ^2^ ^]^ It is estimated that the prevalence of CNS diseases continues to increase, with more than 103 million people to be affected in 2030.^[^ ^3^ ^]^ Despite the bitter truth, the development of CNS disease therapeutics has made little progress for decades. To date, small molecule drugs remain to be the first line of treatments in clinics. For instance, aducanumab, donepezil, rivastigmine, galantamine, and memantine are used to treat AD,^[^ ^4^, ^5^ ^]^ L‐Dopa is applied to manage PD,^[^ ^6^, ^7^ ^]^ and tetrabenazine is utilized to address HD.^[^ ^8^, ^9^ ^]^ Nevertheless, they are not curative but symptomatic treatments to delay disease progression. For glioblastoma (GBM) and other CNS cancers, temozolomide (TMZ) is still the only first‐line drug that is approved by U.S. Food and Drug Administration (FDA). In recent years, immunotherapies, such as modulation of cytokines, vaccinations using monoclonal antibodies and specific antigens, may provide an alternative therapeutic approach, though they are still at the infant stage.^[^ ^10^, ^11^ ^]^ With the explosive development of genome engineering technologies, gene therapy has heralded a new era for improving the treatments of brain diseases.^[^ ^12^, ^13^ ^]^ Compared to classic small molecule‐based therapeutic approaches, gene therapy offers unprecedented leverage in drug development, including the prospect of regulating genes encoding proteins that are deemed undruggable by conventional small molecules, high target specificity, reversible effect, and the potential to be re‐programmed without alternating pharmacokinetics features.^[^ ^14^, ^15^ ^]^ Gene therapy is also linked to immunotherapy and/or cellular therapy as exemplified by the chimeric antigen receptor T cells (CART) therapy, which uses the genetically engineered T cells and becomes the most advanced approach.^[^ ^16^, ^17^ ^]^

The genotyping of many, if not most, CNS diseases has revealed numerous novel therapeutic targets. A number of promising formats that are able to activate or deactivate those targets have been identified, including plasmid DNA,^[^ ^18^, ^19^ ^]^ small interference RNA (siRNA),^[^ ^20^, ^21^, ^22^, ^23^ ^]^ antisense oligonucleotides (AONs),^[^ ^24^, ^25^, ^26^ ^]^ miRNA,^[^ ^27^, ^28^ ^]^ and mRNA.^[^ ^29^, ^30^ ^]^ Additionally, the clustered regularly interspaced short palindromic repeat‐associated protein (CRISPR/Cas)‐mediated genome editing also yielded promising results to modulate genes, which has the potential to revolutionize the treatment of CNS diseases.^[^ ^31^, ^32^, ^33^ ^]^ However, delivery of these genetic therapeutics to the brain still remains challenging owing to several factors: 1) fast clearance of genetic therapeutics in the blood circulation; 2) inadequate cellular internalization; 3) limited specificity and off‐target effect; 4) inability to cross the blood‐brain barrier (BBB). Viral vectors, especially Adeno‐associated viral (AAV) vectors, have recently been applied for the delivery of therapeutic genes to address the gene mutations in CNS diseases.^[^ ^34^ ^]^ In 2019, the FDA approved an AAV‐based gene therapy Zolgensma (AveXis, Inc., Illinois, U.S.) to treat spinal muscular atrophy (SMA) by replacing survival motor mutations protein (SMN).^[^ ^35^ ^]^ It is reported that AAV vectors, such as AAV9, AAV rh.8, and AAV rh.10, not only display a high transfection efficiency, but also exhibit the capability to cross the BBB after intravenous administration.^[^ ^36^, ^37^, ^38^, ^39^ ^]^ Despite these advantages, clinical translation of many virus‐based gene therapies for CNS diseases treatment has been restricted due to safety concerns caused by pre‐existing immunotoxicity, oncogenesis, risks of insertional mutagenesis, and off‐target issues.^[^ ^40^, ^41^, ^42^, ^43^ ^]^

The development of nanocarriers as non‐viral vectors to deliver genetic therapeutics to the CNS is an emerging pharmacotherapeutic strategy to overcome these obstacles. To date, a plethora of nanocarriers have been proposed and examined, including inorganic nanoparticles (NPs) (e.g., gold NPs and iron oxide NPs),^[^ ^44^ ^]^ polymer‐based NPs,^[^ ^45^, ^46^ ^]^ exosomes^[^ ^18^, ^47^ ^]^ and lipid‐based NPs.^[^ ^48^, ^49^ ^]^ Compared to the viral vectors, these nanomedicine‐based gene delivery systems offer various advantages, including increased targeting specificity, prolonged blood circulation, high loading capacity, controllable drug release, and lower immunogenicity.^[^ ^50^, ^51^, ^52^ ^]^ More importantly, the installation of brain‐specific ligands on the nanocarriers enables the delivery of cargoes to the specific diseased sites in the brain.^[^ ^13^, ^53^ ^]^ Nevertheless, several obstacles still exist that hinder their clinical applications. First, acute and long‐term neurotoxicity of NPs remains to be further explored within the broader nanotoxicity paradigm.^[^ ^54^ ^]^ Additionally, the relationship between NPs behavior, such as NP distribution and pharmacokinetics, and disease pathophysiology is poorly studied.^[^ ^55^ ^]^ The capacity and cost‐effectiveness for scaling up production and clinical translation of NPs are also important challenges to be addressed.

**Figure 1:**
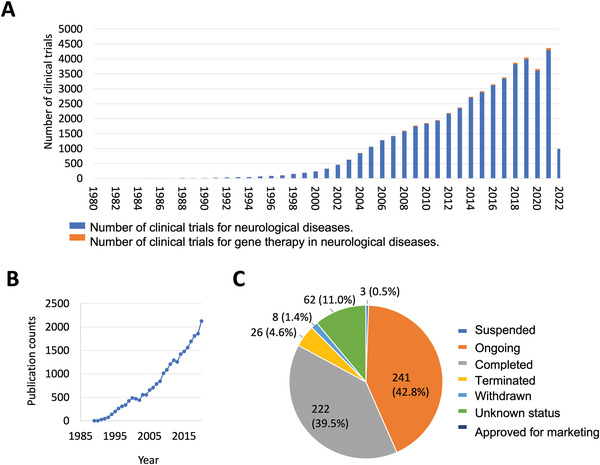
The trend in gene therapy for neurological diseases. A) Number of clinical trials of gene therapy for neurological diseases.^1)^ B) Number of publications in gene therapy for neurological diseases.^2)^ C) Status of clinical trials of gene therapy in neurological diseases.^3)^ Note: 1) Results by February 2022 on ClinicalTrials.gov on the condition of “neurological disease”. The number of gene therapy clinical trials are searched on the condition of “neurological diseases and gene therapy”. 2) Results by February 2022 on Web of Sciences Core Collection by Advance searching: TS = (((gene therapy*) or (siRNA) or (AON*) or (RNA interference) or (CRISPR)) and ((brain disease*) or (neurological disease*) or (neurodegenerative disease*) or glioblastoma or (AD*) or (PD*) or (HD*) or (brain tumor*) or (CNS tumor*) or (CNS disease*) or (CNS cancer*) or (brain cancer*))). 3) Ongoing is defined as “not yet recruiting; recruiting; enrolling by invitation; active, not recruiting, available”.

In order to improve the success rate of nanomedicines in clinical translation, rational designs of gene therapies for brain diseases need to be explored from both therapeutic, and drug delivery perspectives. Of note, the research on CNS diseases and relevant gene therapy has been steadily increasing over the past decades as evidenced by the fast‐growing number of clinical trials (**Figure** **1**A) and publications (Figure 1B). However, the development of gene therapies is still in the incubation period and there is no approved gene therapy for CNS diseases in the clinic so far (Figure 1C). To facilitate the development of this field, an up‐to‐date review of the cutting‐edge potential genetic modulators and CNS gene delivery technologies is thus instrumental and timely. Here, we aim to discuss the recent advancements of gene therapy‐based nanomedicines for CNS diseases, outlining their characteristics, advantages, potential opportunities, and challenges in clinical applications. First, we describe the disease‐implicating genes in major CNS diseases and their therapeutic potentials (**Table** **1**). Second, we highlight the approaches to delivering gene modulators to the diseased brain tissues using nanomedicines, and their potential to cross the BBB. We envisage such a systematic review of nanomedicine approaches will provide cross‐disciplinary insights to enable the realization of advanced gene‐therapies for the treatment of CNS diseases.

**Table 1:** Selected disease‐implicating genes in CNS diseases

	Implicated genes	Mechanisms	Clinical Trials	Ref.
GBM	*IDH*	Regulate glutamine metabolism, glucose sensing, lipogenesis, and cellular redox status.	N.A.	^[^ ^56^ ^]^
	*EGFR*	Regulate cell growth, migration, invasion, angiogenesis, metastasis, and chemo resistance	NCT02844062 NCT03283631 NCT01454596	^[^ ^57^ ^]^
	*FGFR*	Related to glioma growth, vascularization, and invasion	N.A.	^[^ ^58^, ^59^ ^]^
	*TERT*	Regulate telomerases.	N.A.	^[^ ^60^, ^61^ ^]^
	*nc‐RNA*	Regulates proliferation, migration, radio resistance, tumorgenicity.	N.A.	^[^ ^62^ ^]^
AD	*APOE*	Regulate lipoprotein metabolism, A*β* metabolism. Critical in neurotoxicity, tau phosphorylation, synaptic plasticity, neuroinflammation, vascular integrity, glucose metabolism, and mitochondrial function.	N.A.	^[^ ^63^, ^64^ ^]^
AD	*APOE*	Regulate lipoprotein metabolism, A*β* metabolism. Critical in neurotoxicity, tau phosphorylation, synaptic plasticity, neuroinflammation, vascular integrity, glucose metabolism, and mitochondrial function.	N.A.	^[^ ^63^, ^64^ ^]^
	*APP*	Regulate Aβ clearance, amyloid deposition, and neuritic toxicity. Also associated with the complement system.	N.A.	^[^ ^65^ ^]^
	*PSEN*	Regulate Aβ production.	N.A.	^[^ ^66^, ^67^, ^68^ ^]^
	*NGF* ^a)^	Stimulate the function of cholinergic neurons, prevent cell death, and improve memory.	NCT00087789	^[^ ^69^ ^]^
PD	*SNCA*	Regulate α‐Syn production and Lewy bodies’ formation, activate microglial and neuroinflammation.	N.A.	^[^ ^70^, ^71^ ^]^
	*GBA*	Increase α‐Syn aggregation (gain‐of‐function theory), Regulate lysosomal function and substrate accumulation (loss‐of‐function theory).	NCT04127578	^[^ ^72^, ^73^ ^]^
	*NF* ^b)^	Regulate dopamine neuron growth and maintenance	NCT04167540 NCT01621581	^[^ ^74^ ^]^
	*nc‐RNA* ^c)^	Regulate *SCNA* expression and Lewy body formation; Related to mitochondrial dysfunction and oxidative stress, apoptosis, NF expression, and neuroinflammation.	N.A.	^[^ ^75^, ^76^ ^]^
HD	*HTT*	Regulate mitochondrial function, oxidative stress, and transcription.	NCT04120493	^[^ ^77^, ^78^ ^]^
Stroke	*TSPAN2, PMF1‐SEMA4A*	Not understood yet.	N.A.	^[^ ^79^ ^]^
	Nc‐RNA	Regulate apoptosis and inflammation.	N.A.	^[^ ^80^, ^81^, ^82^ ^]^

^a)^ *NGF* represents genes encoding nerve growth factors.

^b)^ *NF* represents the genes encoding neurotrophic factors.

^c)^ nc‐RNA represents non‐coding RNA.

## 2 Disease‐Implicating Genes and Their Therapeutic Potentials

### 2.1 Alzheimer's Disease (AD)

AD is the largest CNS disease. Much of our current understanding of AD neuropathology is based on the accumulation of amyloid *β* (A*β*) in the brain and abnormally phosphorylated tau that contribute to forming neurofibrillary tangles (NFTs).^[^ ^83^ ^]^ In 2021, the FDA approved the first A*β*‐targeting monoclonal antibody, aducanumab, for AD treatment though its therapeutic benefit is still disputed.^[^ ^84^ ^]^ To date, a combination of genome‐wide association studies (GWAS) and experimental studies have identified many putative susceptibility genes that are associated with early onset AD (EOAD) and late onset AD (LOAD).^[^ ^85^, ^86^, ^87^, ^88^ ^]^ Four main pathways have been closely associated with these genes, including lipid metabolism (e.g., *APOE*, *APOC1*, *APOM*, *APOA5*, *ABCA1*), immune response (e.g., *CLU, CR1, RELB*), tau pathway (e.g., *B1N1, CD2AP, SCNA, APOM, APOA5, FERMT2, CASS4, PTK2B*) and amyloid precursor protein (APP) metabolism (e.g., *FERMT2, PICALM*).^[^ ^88^, ^89^ ^]^ A number of common or rare functional variants in certain genes also show suggestive association with AD, such as *APOE*, *CR1*, *TREM2*, *CLU*, *CD33*, *ECHDC3*, and *ACE*.^[^ ^90^ ^]^

Of the discovered genetic risk factors, *APOE* encoding apoliprotein E is the most validated susceptibility gene, which contributes to more than 30% of the genetic variance in LOAD. Prevailing evidence suggests that APOE protein has pleiotropic functions in the CNS, including being the major cholesterol and other lipid carriers.^[^ ^63^ ^]^ In addition, APOE also plays critical role in neurotoxicity, tau phosphorylation, synaptic plasticity, neuroinflammation, vascular integrity, glucose metabolism, and mitochondrial function.^[^ ^64^ ^]^ The differential influence of APOE isotypes on A*β* accumulation and clearance are critically involved in AD pathogenesis. Among the three major alleles encoded by APOE *ε*2, *ε*3, and *ε*4, the *APOE4* allele is said to be the strongest genetic risk factor for LOAD, which is likely to function by increasing amyloid burden in the brain.^[^ ^91^ ^]^ By contrast, *APOE2* allele displays a protective effect against AD and is associated with A*β* clearance.^[^ ^92^ ^]^

In addition to APOE, mutations in three other genes, including *APP* encoding APP, *PSEN1*, and *PSEN2* encoding presenilin 1 and 2, respectively, are also believed to critically contribute to neurodegeneration in AD. Many studies have shown that abnormal processing of APP attributes to AD development. It is reported that the cleavage of APP is mediated by *β*‐site APP cleaving enzyme 1 (BACE1, encoded by *BACE1*). One of the cleavage products A*β*42 is considered as the major amyloidogenic peptide accumulated in the AD brain tissues, whereas another product A*β*40 is counted as the major non‐amyloidogenic peptide.^[^ ^65^ ^]^ Some researchers reported that the *PSEN* mutation may alter the ratio of A*β*42 and A*β*40 and the quality of A*β* species (gain‐of‐function theory), while some hold the opposite view that *PSEN* prevents A*β* generation and its mutations lead to AD (loss‐f‐function theory).^[^ ^66^, ^67^, ^68^ ^]^ Despite this controversy, the potential of manipulating *APP* and *PSEN* mutations, as well as the relative pathways, has been explored.^[^ ^93^, ^94^ ^]^ For example, a significant reduction in A*β*‐associated pathologies and improved learning and memory performance were observed when *BACE1* gene was targeted via CRISPR‐Cas9 nanocomplexes in two AD mice models.^[^ ^95^ ^]^ Notably, *APP*/*PSEN* transgenic cells and animals are generated and broadly used as disease models in AD studies.^[^ ^95^, ^96^, ^97^, ^98^ ^]^

Genes encoding the immune receptors have also been identified as genetic risk factors for AD, including gene *TREM2* encoding triggering receptor expressed on myeloid cells 2. Over the past years, neuroinflammation has been demonstrated to play an important role in the pathogenesis and progression of AD.^[^ ^99^ ^]^ Though A*β* deposits alone can trigger an inflammation that contributes to AD development, the pathophysiology of neuroinflammation is more complicated and involves the activation of different brain cells, particularly the microglia, the resident phagocytes of the CNS.^[^ ^100^ ^]^ During AD progression, microglia are activated to transform from M2, a neuroprotective phenotype, to M1, a neurotoxic phenotype, which secrets a number of inflammatory cytokines and chemokines, such as interleukin‐1*β* (IL‐1*β*), IL‐6, reactive oxygen species (ROS), and tumor necrosis factor *α*.^[^ ^101^, ^102^ ^]^ These mediators largely decrease the ability of microglia to clear A*β*, leading to A*β* deposits which further deteriorate AD progression. The gene *TREM2*, which is exclusively expressed by microglia in the brain, has been found to regulate inflammatory signaling, as well as sustain the proliferation and survival of microglia.^[^ ^103^, ^104^ ^]^ This renders *TREM2* a promising target for AD treatment. A recent study by Wang et al. shows that systematic delivery of nanocomplexes carrying TREM2‐expressing plasmid to microglia significantly enhanced A*β* clearance and improved cognitive performance in AD mice.^[^ ^105^ ^]^ Some other genetic factors, such as CD33, CD36, and toll‐like receptors‐4 (TLR4), have also been found to reduce A*β*‐induced cytokine production and regulate neuroinflammation. These are potential targets to develop gene therapy for AD treatments.^[^ ^106^, ^107^, ^108^ ^]^

In addition to the abovementioned genetic risk factors for AD, the genes that encode neurotrophins have also been indicated as potential targets for AD gene therapy. For example, nerve growth factors (NGF, encoded by *NGF* gene), which are reported to be involved in many physiological functions, including memory enhancement, death prevention, and functional state stimulation of cholinergic neurons, are used as a prototype of the neurotrophin in AD treatment.^[^ ^69^ ^]^ Though functional variants and the biological pathways remain to be further investigated for most of the AD loci, various gene therapy studies have been carried out to target the AD‐implicating genes as detailed in Section 3.

### 2.2 Parkinson's Disease (PD)

PD is the second most common CNS disease, which is pathologically characterized by the loss of dopaminergic neurons in the *substantia nigra* and the appearance of the protein aggregates, named Lewy bodies, in the midbrain.^[^ ^109^ ^]^ To date, Multiple GWAS have identified more than 90 genetic variants contributing to disease risk and progression for early‐onset and later‐onset PD, including rare and common genetic variants, such as *SNCA*, *GBA*, *LRRK2*, *VPS13C*, and *PRKN*.^[^ ^110^, ^111^, ^112^, ^113^ ^]^ Classically, several pathways are hypothesized to be associated with those genes, including lysosomal and endocytosis pathways (e.g., *GBA*, *LRRK2*, *SNCA*, *VPS35*), and mitochondrial damage repair/elimination pathway (e.g., *PARK7* (or DJ‐1), *PRKN*, *PINK1*).^[^ ^114^, ^115^ ^]^ However, the relevance of many genes is still disputed, and their biological functions also remain poorly understood. Here, we focus on a few that are intensively investigated in various studies.

*SNCA* encoding *α*‐synuclein (*α*‐Syn) is the first risk gene discovered in PD. Typically, the accumulation of *α*‐Syn forms toxic oligomers and aggregates within neurons, and eventually manifests as Lewy bodies. It is believed that these Lewy bodies are involved in the microglial activation and neuroinflammation, which further contribute to the pathogenesis of PD.^[^ ^70^, ^71^ ^]^ Therefore, suppressing the expression of the genes related to *α*‐Syn production is one of the main strategies in PD drug development, among which *SNCA* is the most exclusively explored. Multiple mechanisms are reported to be involved in *SNCA* expression regulation, including epigenetic regulation such as DNA methylation and histone post‐translation modification, and genetic regulation.^[^ ^116^, ^117^ ^]^ So far, various gene therapies have been developed to target SNCA and its related pathways, including AONs,^[^ ^118^ ^]^ siRNA,^[^ ^119^ ^]^ short hairpin RNA,^[^ ^47^, ^120^ ^]^ plasmid DNA,^[^ ^121^, ^122^ ^]^ and CRISPR.^[^ ^123^, ^124^ ^]^ These studies showed that such treatments were able to downregulate *SNCA* expression and reduce Lewy body pathology and *SNCA*‐mediated behavioral deficits. In addition, NGF and basic fibroblast growth factor (bFGF) pathways have also been reported to modulate *α*‐Syn transcription regulation, which could be an alternative target for gene therapy.^[^ ^125^ ^]^

*GBA* gene encoding the lysosomal enzyme glucocerebrosidase, is another important and common implicated gene for PD. Generally, mutations in *GBA* are more frequently seen in patients with PD than other genes such as *SNCA*, *LRKK2*, and *PARK2*.^[^ ^126^ ^]^ To date, more than 300 different mutations are reported in *GBA*, including point mutations, frameshift or in‐frame alterations, splice junction mutations, and recombination events with a highly homologous pseudogene downstream.^[^ ^127^, ^128^ ^]^ *GBA* variants have been identified to be linked to cognitive impairment and motor symptoms in PD patients over time.^[^ ^129^, ^130^ ^]^ However, the mechanisms underlying the *GBA* mutations and the pathology of PD remain disputative. Briefly, the gain‐of‐function theory hypothesizes that the mutations in *GBA* result in the increased *α*‐Syn aggregation, while the loss‐of‐function theory attributes *GBA* deficiency to lysosomal dysfunction and substrate accumulation, which subsequently affect *α*‐Syn processing or cause impairment of autophagy.^[^ ^72^, ^73^ ^]^

Similar to AD, genes encoding neurotrophic factors (NF, encoded by *NF* gene) are widely targeted in PD gene therapy development. NFs are required for dopamine neuron growth, which plays a critical role in CNS maintenance.^[^ ^74^ ^]^ Glia cell line‐derived neurotrophic factors (GDNF, encoded by *GDNF* gene), brain‐derived neurotrophic factors (BDNF, encoded by *BDNF* gene), and neurturin (NRTN, encoded by *NRTN* gene) are the three NFs that are most inclusively targeted in gene therapy.^[^ ^131^, ^132^ ^]^ In recent years, genes encoding cerebral dopamine neurotrophic factor (CDNF, encoded by *CDNF* gene) and mesencephalic astrocyte‐derived neurotrophic factor (MANF, encoded by *MANF* gene) have also emerged as new targets.^[^ ^133^ ^]^ Researchers have attempted to externally deliver *NF* genes to the PD diseased site. Indeed, AAV and other viral vectors have been applied to deliver *GDNF*, *BDNF*, and *NRTN* genes since decades ago. Following encouraging preclinical data, several clinical studies which invest AAV‐GDNF and AAV‐NRTN gene therapy in PD patients were conducted.^[^ ^134^, ^135^, ^136^ ^]^ However, the impact of the clinical outcomes remains limited owing to insufficient therapeutic efficacy. Two clinical trials (NCT01621581, NCT04167540) are currently underway to test the potential of AAV‐GDNF expression and no conclusion has been drawn so far. The viral vectors used in these studies are concerned as they may cause immunotoxicity, phenotoxicity, insertional mutagenesis, and transmission of the donated DNA.^[^ ^41^ ^]^ Alternatively, numerous researches are developing non‐viral nanomedicines to deliver *NF* genes, such as liposomes,^[^ ^137^, ^138^ ^]^ plasmid DNA NPs,^[^ ^139^ ^]^ and chitosan NPs,^[^ ^122^ ^]^ which demonstrated neuroprotection effects, showing reduced dopaminergic neuron loss and the improved behavioral metrics in PD animal models. Such approaches may potentially yield clinical benefits in PD therapy, which will be further detailed in Section 3.

In addition to the coding variants at disease‐implicating genes, the non‐coding variants are also commonly seen to increase the risk for PD. A number of non‐coding RNAs (ncRNAs) have been identified to involve in PD pathogenesis via: a) regulating *SCNA* expression and Lewy body formation (e.g., miR‐7, miR‐34b/c, miR‐133b); b) regulating responses to mitochondrial dysfunction and oxidative stress (e.g., miR‐27, miR‐221 and miR‐494, and miR‐205); c) regulating apoptosis (e.g., miR‐126, miR‐342‐3p); and d) regulating NF expression and neuroinflammation (e.g., *NEAT1*, LncRNA‐p21, miR‐153, miR‐7) (reviewed in refs. [75, 76]).Though there have been no attempts targeting these ncRNAs so far, they are thought to be potential new targets for PD diagnosis or gene therapy development.

### 2.3 Brain Tumors

Brain tumors are devastating diseases that account for 2% of all cancers but disproportionately lead to cancer‐related indisposition, morbidity, and mortality around the world in both adults and children.^[^ ^140^, ^141^ ^]^ Over the past decades, multiple studies have been conducted to study the genetic basis of tumorigenesis in both common and rare brain tumors. Gliomas are the most common primary brain tumors in adults, which generally can be classified into glioblastoma (GBM), and low‐grade non‐GBM tumors such as grade II astrocytoma, grade II oligodendrogliomas).^[^ ^142^, ^143^, ^144^ ^]^ Notably, genetic susceptibility to GBM and non‐GBM gliomas is relatively distinctive. To date, GWAS and transcriptional‐wide association studies (TWAS) have identified numerous genetic risk factors associated with GBM (e.g., *EGFR, TP53, FGFR, PTEN, CDKN2A, TERT, TERC*) and non‐GBM glioma (e.g., *VTI1A, MDM4, AKT3, IDH1, TREH, TERT, TMEM25, PHLDB1*).^[^ ^85^, ^86^, ^144^, ^145^, ^146^ ^]^ To some extent, such distinction reflects the different etiologies underlying the diseases and also implicates the different druggable gene targets. Here, we focus on GBM, one of the deadliest CNS tumors, which is the first tumor comprehensively studied by the Cancer Genome Atlas (TCGA).^[^ ^147^ ^]^ Traditionally, GBM was considered either primary or secondary based on clinical observations and histologic evidence.^[^ ^148^ ^]^ Subsequent studies have identified various genome alterations. Three pathways have been found to be critically associated with the frequent genetic alterations, including: 1) Receptor tyrosin kinase signaling (e.g., *PTEN/NF1/PIK3CA, EGFR/PDGFRA*); 2) tumor protein 53 (TP53) signaling (e.g., *CDKN2A, TP53, MDM1/MDM4*); 3) retinoblastoma (RB) signaling (e.g., *CDKN2A/CDKN2C, RB, CD4/CD6*).^[^ ^149^ ^]^ Later, more disease molecular markers have been identified. The 2016 CNS WHO revised GBM classification into *IDH*‐wildtype (≈90% cases) and *IDH*‐mutant GBM (≈10% cases), which display identical histopathology but have different genetic features. Isocitrate dehydrogenase encoded by *IDH* is involved in many cellular metabolic functions, including glutamine metabolism, glucose sensing, lipogenesis, and regulation of cellular redox status.^[^ ^56^ ^]^ Mutations in *IDH*, especially in *IDH1* and *IDH2*, are gain‐of‐function mutations that produce oncometabolite 2‐hydroxyglurate (HG), which competently inhibits enzymes binding alpha‐ketoglutarate (*α*‐KG) and subsequently causes upregulation of *VEGF* and histone methylation.^[^ ^150^ ^]^ Such oncogenic effects of *IDH* mutations are seen to be correlated with other genetic risk factors, including *EGFR*, *TP53*, and *ATRX* mutations, thus leading to tumor progression.^[^ ^56^ ^]^ Correcting mutations in these genes using gene therapy is one strategy to treat GBM.

Genes that encode receptor tyrosine kinases (RTKs) and their downstream signaling pathways are also viewed as the risk variants in GBM progression.^[^ ^57^ ^]^ They have been found to involve in cell growth, migration, invasion, angiogenesis, metastasis, and chemoresistance in GBM (reviewed in ref. [151]). The main RTKs include epidermal growth factor receptor (EGFR, encoded by *EGFR*), fibroblast growth receptor (FGFR, encoded by *FGFR*), vascular endothelial growth factor (VEGFR, encoded by *VEGFR*), insulin‐like growth factor receptor (IGFR, encoded by *IGFR*), platelet‐derived growth factor (PDGF, encoded by *PDGF*) and hepatocyte growth factor receptor (HGFR, known as MET, encoded by *HGFR*).^[^ ^152^ ^]^ Among these, *EGFR* alterations, especially *EGFRvIII*, are the most frequently observed to drive GBM tumorigenesis.^[^ ^153^, ^154^, ^155^ ^]^ A number of preclinical and clinical attempts have developed various strategies to target *EGFR*, including the NPs‐based gene therapy, such as polymeric NPs,^[^ ^156^ ^]^ iron oxide NPs,^[^ ^157^ ^]^ and solid lipid NPs.^[^ ^158^ ^]^ Data from these studies consistently showed that the treatments led to a significant decrease in *EGFR* expression and the inhibition of tumor growth both in vitro (GBM cell/stem cell, spheroids) and in vivo (GMB‐bearing mice). Despite these attempts, successful clinical translation of gene therapies targeting *EGFR* has not been reported yet. Besides *EGFR*, *FGFR* is also an oncogenic RTK gene widely studied, which contributes to glioma growth, vascularization, and invasion.^[^ ^58^, ^59^ ^]^ Yamada et al. attempted to target *FGFR1* by AON, which significantly inhibited *FGFR1* expression and reduced GBM cell growth.^[^ ^159^ ^]^ Attempts are also being made to develop multiple RTKs‐targeted genetic therapeutics for GBM treatment. For example, a team from Sirnaomics Inc. investigated a siRNA cocktail NP (STP‐523) with multiple targets *EGFR*, *VEGF*, and *AGT* encoding O6‐mehtylguanine‐DNA‐methlytransferase for treatment of GBM, and observed enhanced treatment effect compared to the single gene‐targeted siRNA inhibitors.

Mutations in the promoter of *TERT* gene encoding telomerase reverse transcriptase is another strong factor associated with GBM risk, which was frequently observed in GBM especially in adult patients.^[^ ^60^, ^61^ ^]^ The role of *TERT* in GBM oncogenic mechanisms has not been fully understood yet. Various ideas have been suggested. One hypothesis is that such mutation leads to *TERT* overexpression and facilitates telomerase reactivation, which overcomes replicative senescence.^[^ ^160^ ^]^ Interestingly, *EGFR* mutations were observed to highly appear in GBM with *TERT* mutations.^[^ ^60^ ^]^ Several preclinical studies are developing gene therapies that target *TERT* and its downstream pathways. A recent proof‐of‐concept study showed that correcting mutated *TERT* promoters using CRISPR‐Cas9 approach reduced *TERT* expression, induced senescence, and inhibited proliferation in several human GBM cell lines, suppressed tumor growth in orthotopic GBM xenograft model.^[^ ^161^ ^]^ GBM tumor inhibition was also achieved by combination of TMZ treatment and CRIPSR to delete genes encoding transcript factor GABPb1L, which bound and activated the mutant *TERT* promoter.^[^ ^162^, ^163^ ^]^ Furthermore, telomere aberration in brain tumors can also result from mutations in *ATRX* encoding telomere‐binding protein alpha thalassemia/mental retardation syndrome X‐linked, which exclusively occurred along with *TERT* promoter mutations, and/or *DAXX* (encoding death‐domain associated protein).^[^ ^60^, ^164^, ^165^ ^]^ These genes could also potentially serve as targets for the development of GBM genetic therapeutics.

Many miRNA have been demonstrated associating with cell proliferation, differentiation, apoptosis, migration, and angiogenesis in GBM. So far the alterations of many miRNA have been identified as potential GBM biomarkers, including miR‐21, miR‐221/222, miR‐124, miR‐128, miR‐7, miR‐137, miR‐181, miR‐218, miR‐148a, and miR‐10b.^[^ ^62^ ^]^ They are involved in regulating the expression of critical GBM‐driving genes such as *EGFR* (e.g., miR‐7), *MGMT* (e.g., miR‐181d, miR‐198), and *BCL2* (e.g., miR‐7). Among these, miR‐21 is found to be consistently overexpressed in GBM, facilitating GBM progression by regulating EGFR, p53, PTEN, MMP, TFR‐*β* pathways. The therapeutic efficacy of silencing miR‐21 was confirmed by using AON delivered by polymer NPs,^[^ ^166^, ^167^ ^]^ exosomes,^[^ ^168^ ^]^ dendrimers,^[^ ^169^ ^]^ and peptides.^[^ ^170^ ^]^ Besides, nucleic acids have also been developed to target miR‐23a, mi92b, miR‐155‐5p, and miR‐221‐3p, which induced glioma cell apoptosis and suppressed cell growth.^[^ ^171^, ^172^, ^173^ ^]^ As more GBM‐related miRNA are being discovered and their implications to the pathology of GBM are being clarified, therapies that modulate miRNA will become more promising for GBM treatment.

Therapeutic approaches have been also directed to regulate other genes involved in epigenetic alterations, including DNA methylation, histone modification, and chromatin structural aberration, which are commonly seen during gliomagenesis. A compelling example is a DNA repair enzyme O^6^‐methylguanin DNA methyltransferase (MGMT, encoded by *MGMT* gene), which has been identified to counteract the cytotoxic effect of alkylating chemotherapy such as TMZ. Methylation of CpG island in *MGMT* promoter area has been found to cause epigenetic silence of *MGMT*, which was observed in 45% of GBM patients.^[^ ^174^ ^]^ Thus, silencing *MGMT* and its subsequent loss of protein expression could serve as a promising therapeutic target. For instance, studies show that siRNA and AON against *MGMT* increased sensitivity and cytotoxicity of TMZ treatment in both in vitro and in vivo GBM tumor models.^[^ ^175^, ^176^ ^]^

### 2.4 Huntington's Disease (HD)

HD is an inherited progressive CNS disease characterized by a movement disorder, cognitive decline, and behavioral and psychiatric symptoms.^[^ ^177^ ^]^ Different from other CNS diseases, HD is caused by a monogenic mutant, a CAG trinucleotide repeat expansion, in *HTT* gene encoding Huntington protein, which has been found to determine the timing of HD onset.^[^ ^178^, ^179^ ^]^ Numerous studies indicated that toxicity resulting from HTT mutant, CAG RNA toxicity, loss of function of HTT, polyQ protein misfolding, and aggregation could affect several cellular processes, such as mitochondrial dysfunction, oxidative stress, transcriptional deregulation, thus contributing to HD pathogenesis.^[^ ^77^, ^78^ ^]^ Despite these findings, so far there is no curative drug but symptomatic drug for HD treatment.

Owing to its monogenetic nature, most HD drug development studies aim to suppress the production of the mutant *HTT*. Interfering the causative *HTT* gene expression by genetic modifier or gene editing technology is one of the key approaches. Numerous studies have been carried out to target mutant *HTT* via siRNA,^[^ ^21^, ^180^, ^181^ ^]^ AON,^[^ ^182^ ^]^ microRNA,^[^ ^27^, ^28^ ^]^ and CRISPR.^[^ ^183^ ^]^ Preclinical studies report that such an approach could achieve *HTT* silencing both in vitro and in vivo. The promising data have been translated into several clinical studies. In 2019, a phase 1/2a clinical trial reported that an AON construct targeting *HTT* gene was capable to reduce mutant *HTT* in cerebral spinal fluids in patients at early onset of HD.^[^ ^184^ ^]^ Currently, two phase 3 trials (NCT03761849, NCT03842969) are being undertaken to evaluate the safety, efficacy, and tolerability of AON‐based drugs in HD patients.

In addition to the *HTT* gene, several genetic modifiers encoding proteins that are associated with HD through different mechanisms have been identified recently, such as Nme1 (suppressing) mutant *HTT*),^[^ ^185^ ^]^ NF (providing) neurotrophic support,^[^ ^186^ ^]^ CYP46A1 (affecting brain cholesterol metabolism),^[^ ^187^ ^]^ and TLR4/TREM2 (affecting inflammation).^[^ ^188^ ^]^ Targeting these genes via gene therapy led to decreased *HTT* aggregation and improved motor performance in HD animal models, which could be potential therapeutic targets for HD drug development.^[^ ^185^, ^186^, ^187^, ^188^ ^]^

### 2.5 Stroke

Stroke is the second largest leading cause of mortality and disability globally, and the overall burden of stroke remains high.^[^ ^189^, ^190^ ^]^ There are two subtypes of stroke, including ischemic stroke, which is mainly caused by brain infarction (accounting for about 80%), and hemorrhagic stroke, which results from leaky bleeding (accounting for 20%).^[^ ^191^ ^]^ Metabolic risks (e.g., high blood pressure, body‐mass index, plasma glucose, total cholesterol), and behavioral factors (e.g., smoking, poor diet, low physical activity) attribute the most to risk factors of both stroke types.^[^ ^190^ ^]^ Compared to other types of brain complications mentioned, less genetic factors are found in the disease mechanism of stroke. Multiple GWAS have found about 32 risk loci related to stroke and stroke subtypes (e.g., *TSPAN2*, *PMF1‐SEMA4A*, *CASZI*, *WNT2B*) and 18 associated pathways, including various cardiac pathways, muscle‐cell fate commitment, and nitric oxide metabolic process.^[^ ^79^ ^]^ Despite these findings, there has been no reported gene therapy that targets these risk loci so far. Instead, majority of current gene therapies target genes which encode the proteins involved in the pre‐ or post‐ stroke process, such as hypoxia‐induced proteins (HIF‐1),^[^ ^192^ ^]^ neuroinflammation‐induced proteins,^[^ ^193^, ^194^ ^]^ apoptosis‐related proteins (e.g., HDAC2, HO‐1),^[^ ^195^, ^196^, ^197^ ^]^ microglial neurotoxicity‐related protein (e.g., C3),^[^ ^198^ ^]^ neuron loss‐related protein (e.g., NeuroD1),^[^ ^199^ ^]^ and atherosclerosis‐related protein (e.g., CD47).^[^ ^200^ ^]^ The brain‐protection effect shown in these studies indicates that these genes may serve as beneficial targets for gene therapy development.

A number of non‐coding RNA (ncRNA), such as miRNA and circular RNA, have also been reported to involve in the biological processes and attribute to the pathogenesis of stroke, such as miR‐17‐92, miR‐195, miR‐152, mi‐124 miR‐130, miR‐381‐3p, miR‐652, and circularSCMH1.^[^ ^80^, ^82^, ^201^, ^202^, ^203^ ^]^ These ncRNAs could potentially be applied as prognostic, diagnostic, and therapeutic biomarkers of stroke in the future. Multiple studies reported that targeting specific miRNA by genetic modulators could sufficiently alleviate apoptosis and inflammation during stroke, reduce the injured brain volume, and enhance neuroplasticity and functional recovery in stroke models.^[^ ^80^, ^81^, ^82^ ^]^ These promising preclinical results highlight the potential of gene therapy for stroke prevention and post‐stroke management, though relatively limited attempts have been tested in clinical trials up to date. Indeed, given that the therapeutic window of stroke is narrow (few hours), especially in acute ischemic stroke, the application of gene therapy may not be suitable in some circumstances.

## 3 Gene Therapy for CNS Diseases

**Figure 2:**
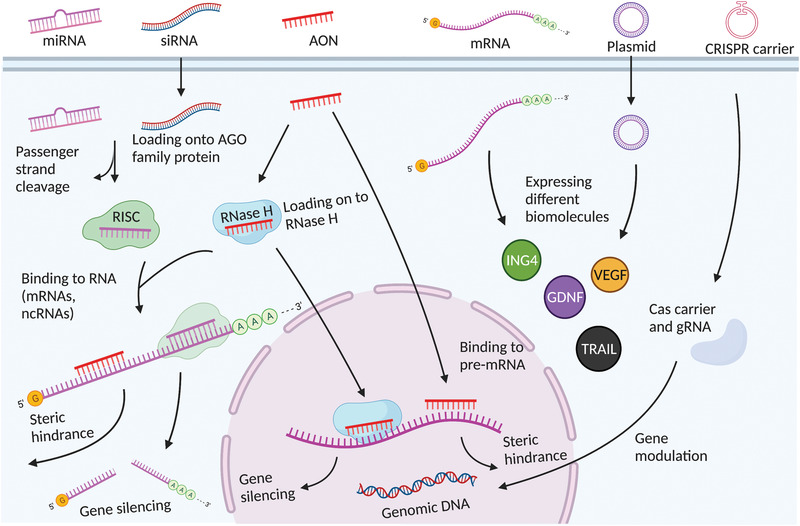
Schematic representation of gene therapies involved in the main CNS diseases. Various gene therapies, including miRNA, siRNA, AON, mRNA, plasmid DNAs, and CRISPR‐Cas, are developed to target and modulate the expression of the CNS disease‐causing genes. Drawn by BioRender.

Gene therapies have been widely investigated in clinical and preclinical studies to treat brain diseases over the past decades. Since the mechanisms of each gene therapeutic approach are different, they largely determine the principles of nanomedicine design. In this section, we summarize the current gene therapies involved in the main CNS diseases and their specific mechanisms (**Figure** **2**).

### 3.1 RNA Interference

RNA interference (RNAi), also known as RNA silencing or post‐transcriptional gene silencing (PTGS), is one of the promising therapeutics in brain disease treatment. Exogenous nucleic acids can be experimentally engineered into cells, which interfere with the expression of disease‐causing genes and thus regulate specific protein production. Historically it was first employed by Fire et al. in 1998.^[^ ^204^ ^]^ From then on, many studies have been carried out to explore the potential targets in various diseases that are “undruggable” by classical small molecule‐based drugs.^[^ ^15^, ^205^, ^206^ ^]^ Small non‐coding RNAs (ncRNAs) are short genes typically consisting of 20–30 nucleotides and featuring in their association with Argonaute family proteins (AGO family proteins), which guide small ncRNA to their regulatory targets. Small ncRNA suppresses the undesired genetic materials and transcription in eukaryotes. By regulating the gene expression, they protect the genome from being harmed by internal and external threats. Considering the difference in biogenesis, modes of target regulation, and biological pathways that they regulate, small ncRNA are mainly classified into three types, including microRNA (miRNA), small interference RNA (siRNA), and PIWI‐interaction RNA (piRNA). Nevertheless, several studies have shown that, regardless of their different features, exclusive collaboration and competition exist among these small RNA pathways.^[^ ^207^ ^]^ However, they are generally suffering from fast clearance, innate immune response, and degradation by enzyme (e.g., RNase).^[^ ^208^ ^]^

#### 3.1.1 miRNA

miRNA are small single‐stranded ncRNAs (19–25 nucleotides) that abundantly existed in many species ranging from human, animals, plants, and virus to protists. They are naturally produced by processing the long hairpin RNA and require a ribonuclease III enzyme, dicer. More than 60% of protein‐coding genes in humans have at least one conserved domain and a number of non‐conserved domains for miRNA binding.^[^ ^209^, ^210^ ^]^ miRNA are critical in many developmental and pathological processes in the body, including differentiation, apoptosis, cell proliferation, embryonic development, stem cell renewal, stress response, and metabolism.^[^ ^211^ ^]^ The regulation of miRNA and/or dysregulation of miRNA has been found to be associated with a variety of human diseases such as cancer, cardiovascular diseases, diabetes, chronic hepatitis, AIDS, and CNS diseases.^[^ ^212^ ^]^ Generally, one particular type of miRNA can target multiple mRNAs in the cytosol, which will be then loaded onto RNA‐induced silencing complex (RISC), one of the AGO family proteins that can degrade the mRNAs.^[^ ^207^ ^]^ Consequently, these miRNA either trigger direct degradation of the targeted mRNA or suppress their translation, both of which typically result in the interruption of specific protein production. In some cases, suppressing the expression of pathogenic miRNA (e.g., miR‐134, miR‐128) is a therapeutic approach to help restore neurological functions.^[^ ^210^, ^213^, ^214^, ^215^ ^]^

Surprisingly, compared to other tissues, the brain has more distinct miRNA expression. It has been seen throughout the brain, including neural stem cells (NSC), germ cells, and fetal brain.^[^ ^216^ ^]^ Considering their influence on neurogenesis, neuronal maturation, and brain development, miRNA are hypothesized to associate with many CNS disorders such as AD, dementia, fragile X syndromes, Rett, cancer, and other neuropsychiatric diseases, including depression, schizophrenia, and drug addiction.^[^ ^217^ ^]^ Some specific miRNA have been identified in the development and maintenance of healthy neurons. For example, miR‐153, highly expressed in mouse and human brain, was shown to be important in adult neurogenesis by regulating the Notch signaling pathway.^[^ ^218^ ^]^ It is also shown that miR‐212 inhibits early neurogenesis by down‐regulating the expression of Methyl‐CpG Binding Protein 2.^[^ ^219^ ^]^

The critical role in neuronal functions renders miRNA potential targets to develop therapeutics for CNS disease. For example, miR‐31 expression level was found to be reduced in AD patients.^[^ ^220^ ^]^ A study led by Barros‐Viegas et al. intracranially injected miR‐31‐carrying lentivirus into the hippocampus of AD triple‐transgenic (3xTg‐AD) mice, which resulted in a simultaneous reduction of BACE1 mRNA levels as well as APP production. And these mice had significant cognitive improvement.^[^ ^221^ ^]^

The overexpression of some miRNA has been found to lead to neurological disfunctions. Using anti‐miRNA to knock down these disease‐causing miRNA is another approach to treat CNS diseases, which works primarily by introducing a steric hindrance to the miRNA.^[^ ^222^ ^]^ For example, two potent onco‐miRNA, namely miR‐21 and miR‐10b, were identified to be overexpressed in many GBM patients, causing poor survival, rapid proliferation, metastasis, and resistance to TMZ treatment. Malhotra et al. systemically delivered two antisense microRNAs (antagomiRs), antagomiR‐21, and antagomiR‐10b, via the cRGD‐tagged PEG‐PLGA NPs into a xenograft GBM mouse model. They not only found these antagomiRs enhanced responses of the cultured GBM cells to TMZ treatment, but also demonstrated that a lower dose of TMZ was required for killing tumor cells in xenograft GBM mice.

#### 3.1.2 siRNA

RNA knockdown can also be achieved by small interference RNAs (siRNAs). siRNA (≈21–23 nucleotides with 2 nucleotide 3’ overhangs) are ncRNAs naturally produced from long double‐stranded RNA structures mediated by Dicer.^[^ ^223^ ^]^ Sharing many similarities, siRNA and miRNA are both short duplex RNA molecules targeting mRNAs and inhibit the protein expression via PTGS of target mRNA transcripts. However, siRNA usually targets only one specific mRNA whereas miRNA can simultaneously target multiple mRNAs.^[^ ^224^ ^]^ siRNA are typically classified into two types, endogenous siRNA (endo‐siRNA) and siRNA derived from exogenous sources (exo‐siRNA).^[^ ^225^ ^]^ Both of them make use of AGO family proteins to form RISC to catalyze cognate gene degradation.^[^ ^226^ ^]^ A number of endogenous siRNA have been identified to regulate the biological functions such as proteostasis,^[^ ^227^ ^]^ embryonic development,^[^ ^228^ ^]^ and synaptic plasticity.^[^ ^229^, ^230^ ^]^ In 2001, Elbashir et al. first found that the introduction of exo‐siRNA was also able to induce PTGS and subsequent gene silencing effects.^[^ ^231^, ^232^ ^]^ Since then, several studies have been carried out to introduce synthetic, double‐stranded exo‐siRNA, as well as molecules of double‐stranded endo‐siRNA‐like features, into the diseased tissues to achieve the therapeutic effects.^[^ ^233^, ^234^ ^]^

For therapeutic applications, siRNA are typically designed based on the target genes. Various rules have been studied to improve their rational design.^[^ ^235^, ^236^, ^237^ ^]^ For example, Yuan et al. found that the performance of the siRNA would not be compromised but instead show improved target specificity when they were asymmetrically structured.^[^ ^238^ ^]^ Several studies also reported that the content of guanine (G) and cytosine (C) had a significant impact on the siRNA candidates, with the optimal G/C content in the low to medium range (30–64%).^[^ ^239^, ^240^, ^241^ ^]^ In addition, avoiding immune stimulatory motifs like “uracil (U)GGC” and “UGU” will improve the targeting effect of the siRNA.^[^ ^242^, ^243^ ^]^ Besides, other factors have been also found to impact the activities of siRNA, such as orientations of siRNA, target site accessibility, and projection of some substituent groups.^[^ ^240^, ^244^ ^]^ Indeed, with the development of computational tools, the open‐source genomic sequence database, and disease pathogenesis, the selection of therapeutic siRNA candidates has been facilitated in the past decade.^[^ ^245^, ^246^ ^]^ Indeed, they can be designed to target nearly any disease‐related genes.

The improved rational designs have been widely employed to develop siRNA‐based therapies for CNS diseases.^[^ ^208^ ^]^ For instance, Wang intravenously injected nanocomplex contained *BACE‐1*‐targeting siRNA to an AD mouse model. These siRNAs efficiently knocked down *BACE‐1*, which improved the cognitive function of AD mice.^[^ ^247^ ^]^ Spencer et al. intravenously injected a siRNA‐loaded nanocomplex that targeted *α*‐syn protein into a PD mouse model, which triggered a significant reduction of *α*‐syn in the brain of the tested mice.^[^ ^22^ ^]^ siRNA have also been designed to target oncogenes of GBM to aid cancer cell killing. For example, Li et al. designed siRNA against the oncogene called human hyaluronan‐mediated motility receptor (HMMR)‐antisense RNA1 (AS1) or HMMR, and transfected several GBM cell lines with these siRNAs. They found that the treated cancer cells had a significant reduction of *HMMR* expression, which inhibited the cell migration and invasion, and suppressed GBM cell growth both in vitro and in vivo.^[^ ^248^ ^]^ Besides, siRNA was designed to target disease‐causing genes for HD and stroke treatment. For example, Save et al. showed that more than 50% of *HTT* mRNA was reduced by siRNA delivered by chitosan NPs in different brain regions of HD mice after intranasal administration.^[^ ^21^ ^]^ Another study attempted to knock down genes expressing nuclear factor (NF)‐*κ*B p65 in stroke mice by systemically injecting curdlan NPs loaded with the targeting siRNA, which increased the neuron density and alleviated the nuclear pyknosis and neuronal necrosis in the brain.^[^ ^249^ ^]^ In a nutshell, siRNA has been used to specifically target a wide range of biomarkers in CNS diseases. Regardless, many challenges still need to be addressed. For example, cautions should be taken to avoid passenger strand (or sense strand) base‐pairing with the unwanted mRNAs.

#### 3.1.3 piRNA

PIWI‐interacting RNA (piRNA, 26–32 nucleotides) are non‐coding small RNA that is derived from single‐stranded precursor RNA. The production of piRNA is usually mediated by Zucchini, an endonuclease protein, and other unknown trimming enzymes, instead of RNase III proteins. piRNA regulates genome integrity of germline cells by suppressing transposons via binding to PIWI subclade of AGO family.^[^ ^250^ ^]^ Though piRNA are thought to be mainly expressed in germline and somatic cells, there is growing evidence that piRNA also expresses and functions in neurons, especially Aplysia neurons.^[^ ^251^ ^]^ It is also found that the piRNA is highly expressed in mouse Hippocampus tissues, which suggests that piRNA plays an important role in memory and learning.^[^ ^252^ ^]^ The precise mechanism of piRNA remains enigmatic, but it has been identified that the loss of PIWI proteins can lead to brain disorders and neurodegenerative dysfunctions like schizophrenia and Rett syndrome.^[^ ^253^, ^254^ ^]^ In 2019, Jain and Stuendl et al. found that the level of piRNA piR_019324 was lowered in AD patients while two piRNA piR_019949 and piR_020364 were upregulated in AD patients.^[^ ^255^ ^]^ Nevertheless, none of these piRNA is significantly correlated to the AD biomarkers pTau and A*β*40, and the underlying mechanism is still elusive. Recently several piRNA (e.g., piR‐9491 and piR‐2488) have also been identified to be associated with GBM disease development.^[^ ^256^ ^]^ Regardless, up to date, no specific gene therapy targeting piRNA for CNS diseases has been demonstrated. Most of the works still focus on discovering the piRNA as the current stage. With further understanding, piRNA might serve as potential targets in the future.

### 3.2 Antisense Oligonucleotides

Antisense Oligonucelotides (AONs) are synthetic, single‐stranded nucleic acids (16–22 nucleotides) that can suppress expression level of a specific RNA (e.g., siRNA, mRNA, pre‐mRNA, etc.), and modulate the protein translation.^[^ ^257^ ^]^ Although not expressed in the cell naturally, AON can achieve good cell penetrating ability with rational designs, such as backbone‐modification (e.g., N‐acetylgalactosamine (GalANc)) and addition of cell‐penetrating peptides (CPPs).^[^ ^258^, ^259^ ^]^ AON can regulate gene and protein expression via various pathways. Once interacting with cognate genes, they can directly degrade the target transcript by interacting with RNase H1. Different from AGO family proteins, RNase H1 actively exists in both cell cytosol and cell nucleus. Therefore, instead of only targeting mature mRNAs like siRNA, AON can target exons and introns of pre‐mRNAs and/or lncRNA in cell nucleus, as well as mature mRNAs in cell cytosol, both of which enable gene silencing effects. AON can also be designed to block the expression of target genes without triggering the transcript degradation, which is usually termed as steric block AON. They have high affinity but lack RNase H1 competence, thus disturbing the transcript RNA‐RNA and/or RNA‐protein interactions. Such steric block AON is widely used to selectively exclude or retain a specific exon (exon‐skipping and exon‐inclusion, respectively). They manipulate pre‐mRNA splicing by masking a splicing signal so that it could escape from the spliceosomes.^[^ ^260^ ^]^ Indeed, the same technology has already been used for splice corruption to increase the expression of beneficial protein isoforms or decrease the expression of harmful ones. So far this kind of splice‐switching AON has been approved by FDA for several diseases, including Eteplirsen (2016) and Golodirsen (2019) for Duchenne muscular dystrophy (DMD), and Nusinersen (2016) for spinal muscular atrophy (SMN2).

The design of the AON determines the mechanism of action of AON. Sequence selection and structural modification are the two aspects governing the mechanism of action. Unlike siRNA, the single‐stranded AON does not require asymmetric strand design. Similarly, various rules have been explored to design AON for better therapeutic effects. For example, the sequence of AUG is often included in the AON to block the translation, where the translation initiates, or to block the RNA. In such cases, backbone modifications are usually involved to avoid the recruitment of RNase H. The structural modification of AON will be further discussed in the following section. For splicing alternation, many factors should be considered during AON design, such as the selection of target sites, length, chemistry, and melting temperature versus RNA strands.^[^ ^261^, ^262^ ^]^ Various computational tools for screening the potential AON candidates have been developed, such as Pfizer RNAi Enumeration and Design tool (PFRED).^[^ ^263^, ^264^ ^]^ Regardless, the design of efficacious AON for splicing modulation is still challenging because the mechanism of mRNA splicing is highly complex.^[^ ^262^, ^265^ ^]^

AON that acts through various mechanisms has been developed to treat different CNS diseases. For example, DeVos et al. identified an AON that targeted and degraded tau mRNA.^[^ ^266^ ^]^ They demonstrated that this AON can selectively reduce the tau mRNA and tau proteins in mouse AD model as shown by qRT‐PCR and Western blot, respectively. The lifespan of the mice was also extended. AON was also designed to suppress the expression of pathogenic genes. In a study by Ward et al., an AON was developed to block the exon‐intron junctions in HeLa cells, which reduced the translation of *STAT3* gene by 60%, a disease‐driving gene in both AD and PD.^[^ ^267^, ^268^, ^269^ ^]^ In addition to gene degradation and blocking, AON was also designed for exon‐skipping purposes as demonstrated by Mogilevsky's studies. They first identified that the gene *MKNK2* encoding the kinase Mnk2 could be alternatively spliced into two isoforms, namely *MnK2a* and Mnk2b, where the former isoform suppressed tumor growth whereas the latter isoform promoted the tumor progression.^[^ ^270^ ^]^ Following these findings, they specifically designed an AON that induced a strong switch in the alternative splicing of *MnK2*, promoting the expression of *MnK2a* while suppressing the expression of *MnK2b*.^[^ ^271^ ^]^ Such treatment significantly sensitized GBM cells to chemotherapy as well as inhibited tumor development in GBM mice. It is noted that most of these studies directly injected AON into the testing animals, in which large doses of AON were needed for the therapeutic effect. This introduces a high risk of side effects, and also increases the cost.

### 3.3 Plasmid DNA

Plasmid DNA is circular dsDNA that can be replicated independently from the host's chromosomal DNA. They naturally exist in bacteria and provide benefits such as conferring antibiotic resistance and degradation. Plasmid DNA is known to have sizes ranging from 1 to 500 kilobase pairs (kbp).^[^ ^272^, ^273^ ^]^ Studies suggested that the transfection efficiency of plasmid DNAs was not affected when the size was up to 20 kbp.^[^ ^274^ ^]^ Compared to viral carriers, expressing the genetic materials of interest continuously in host cells through plasmid DNA has a lower chance of causing safety complications such as insertional mutagenesis and immunogenicity. Therefore, many studies have been conducted to deliver therapeutic plasmid DNA to treat various diseases such as hepatitis and muscular dystrophy.^[^ ^275^, ^276^ ^]^

Efforts have also been made to deliver plasmid DNA expressing proteins related to CNS disease, such as the abovementioned NF (e.g., GDNF), tumor inhibitor of growth 4 (ING4), tumor necrosis factor‐related apoptosis‐inducing ligand (TRAIL), and angiogenic factors (e.g., VEGF).^[^ ^49^, ^277^, ^278^, ^279^ ^]^ For instance, Aly et al. intranasally injected GDNF‐encoding plasmid (p*GDNF*) into a rat model of PD. Behavioral test and TH‐immunostaining showed improved motor functions and dopaminergic fiber densities, which demonstrated the in vivo therapeutic efficacy of p*GDNF* for PD treatment.^[^ ^280^ ^]^ Wang et al. intravenously injected ING4‐encoding plasmid complexed with an interleukin‐6 receptor binding I6P7 peptide and succinoyl tetraethylene pentamine (Stp)‐histidine (I6P7‐Stp‐His/DNA) to glioma‐bearing mice, which significantly prolonged the survival rate of these mice.^[^ ^277^ ^]^ In addition, supplementation of angiogenic factors via plasmid DNA provides another strategy for alleviating tissue impairment after ischemic stroke. Wang et al. intravenously injected VEGF‐encoding plasmid (*pVEGF*) into a mouse model of stroke with the aid of ultrasound‐targeted microbubble destruction (UTMD). This combined plasmid and ultrasound treatment increased vessel density and reduced apoptosis in the treated mice, confirming the protective effects of *pVEGF*.^[^ ^281^ ^]^ Li et al. injected AD mice with plasmid DNA encoding N eprilysin (NEP), an enzyme that can degrade A*β* fibers. After one‐month of treatment, these mice showed improved cognition.^[^ ^282^ ^]^

### 3.4 Messenger RNA

Messenger RNA (mRNA) is a single‐stranded coding RNA that consists of genetic information for translating proteins. Endogenous mRNA originating from the nucleus first exists as precursor mRNA (pre‐mRNA), which contains coding and noncoding sequences (exons and introns). Pre‐mRNA is then processed to mRNA which is deprived of introns.^[^ ^283^ ^]^ The average length of mRNA is around 3 kbps and no more than 15 kbps.^[^ ^284^, ^285^ ^]^ When compared to plasmid DNA, the delivery hurdles of mRNA to the cell are less severe because 1) mRNA does not need to be delivered to the nucleus and 2) mRNA is smaller than plasmid DNA. Similar to plasmid DNA, mRNA will then be translated to therapeutic proteins in the cytosol without modifying the genomic DNA.^[^ ^283^ ^]^ Thus, unlike gene editing approach, synthetic mRNA is a very useful alternative exogenous therapeutic approach to reversibly alter the protein expression in the CNS where tunable production of proteins is possible by changing the mRNA sequence to be expressed. Also, mRNA expression carries a lower risk of carcinogenesis and mutagenesis than gene editing. However, the clinical application of mRNA as a therapeutic tool is limited by its instability and immunogenicity after mRNA transfection.^[^ ^286^, ^287^ ^]^ A transfection reagent is usually required for mRNA delivery to the cells, similar to plasmid DNA. During the pandemic, mRNA‐based vaccines have become one of the major vaccination methods used globally.^[^ ^288^ ^]^ BioNTech/Pfizer and Moderna use lipid‐based NPs (LNPs) as the transfection agent to deliver the mRNA into the cytosol.^[^ ^289^ ^]^

Nevertheless, mRNA and plasmid DNA are more difficult to be delivered to the CNS compared to AON or miRNA. To cross the BBB or blood‐brain tumor barrier (BBTB) and enter the neurons for implementing their desirable therapeutic outcomes,^[^ ^284^ ^]^ the mRNA and plasmid DNA need to overcome more severe size and charge obstacles than AON or miRNA. For example, Peng et al. studied the efficiency of different commercially available transfection agents by intracranially injecting them with therapeutic TRAIL mRNA into mouse models with glioma. They found that TransIT‐mRNA kit showed the highest TRAIL expression in mice.^[^ ^290^ ^]^ Nanomedicines can also serve as the transfection agent to deliver mRNA by an intracerebroventricular administration of Lipid NPs (LNPs) encapsulating luciferase mRNA. Luminometry revealed that the LNPs successfully transfected the cells in the mouse brains.^[^ ^30^ ^]^ Nanomedicines have also been used to deliver and express mRNA via less invasive administration routes. For instance, Dhaliwal et al. demonstrated that, by intranasally injecting cationic liposomes complexed with GFP mRNA, GFP can be expressed inside the neurons of CD‐1 male mice as evidenced by ex vivo fluorescence imaging of the mouse brain.^[^ ^291^ ^]^

Interestingly, peripheral transfection of mRNA can also support the treatment of brain diseases. Olson et al. demonstrated that peripherally transfecting T cells with granulocyte‐macrophage colony‐stimulating factor (Gm‐csf) mRNA into mouse models of PD can restore the population and function of regulatory T cells (Treg) and trigger anti‐inflammatory and neuronal responses. Flow cytometry data showed increased Treg numbers, and immunohistochemistry of the mouse brain sections had reduced microglial cells and increased tyrosine hydroxylase (TH) positive dopaminergic neurons.^[^ ^292^ ^]^ Extracellular vesicles can also serve as a carrier for mRNA for delivery to the brain. For instance, Kojima et al. implanted cell‐derived exosomes subcutaneously to deliver therapeutic catalase mRNA to reduce neurotoxicity and neuroinflammation in mouse PD models. Specifically, the authors engineered HEK293 cells to produce exosomes that were packaged with the mRNA cargo for delivery to the cell cytosol in the brain, leveraging previous observations that exosomes can cross the BBB. Upon subcutaneous implantation of the exosome‐generating cells, the authors showed a significant reduction in markers that were associated with neuroinflammation (like Glial fibrillary acidic protein (GFAP) and CD11b).^[^ ^293^ ^]^ In short, many different strategies for mRNA‐based treatments for the brain are now under active investigation.

### 3.5 Long Non‐Coding RNA (lncRNA)

In addition to small non‐coding RNAs mentioned above, long non‐coding RNAs (lncRNA), the transcripts of more than 200 nucleotides that are not translating into proteins, also broadly exist in human genomes. Though generally expressed less compared to mRNAs, numerous lncRNA are found to function diversely in many cellular contexts and biological processes, including transcriptional regulation, mRNA stability modulation, and post‐translational modification.^[^ ^294^ ^]^ Some lncRNA has been actually reported to encode for small proteins. Generally, the expression of lncRNA follows tissue‐specific patterns. Surprisingly, an estimated 40% of lncRNA transcripts are expressed in the brain, which also displays a spatiotemporally specific expression profile.^[^ ^295^ ^]^ Some lncRNA are found to play critical roles in CNS development, plasticity, and brain disease progression. For example, lncRNA‐LINCO1116 was shown to upregulate in glioma cells and involved in cell proliferation, metastasis, and tumor angiogenesis.^[^ ^296^ ^]^ This sheds a light on designing therapeutic strategies for CNS disease treatments. One of the approaches is to supplement neuroprotective lncRNA. For instance, Wu et al. found that the pre‐injection of lncRNA‐N1LR‐contained AAV could inhibit p53 phosphorylation, which reduced neuronal loss in middle cerebral artery occlusion in a rat model of ischemic stroke.^[^ ^297^ ^]^ Regardless, the precise functions and mechanisms of most lncRNA largely remain to be further understood.

### 3.6 CRISPR‐Cas

The systems of clustered regularly interspaced short palindromic repeats (CRISPR) and CRISPR‐associated protein (Cas) are well known as genome‐engineering technologies that allow precise gene insertion, deletion, expression promotion, or suppression.^[^ ^298^ ^]^ In 2020, the Nobel prize was awarded to Emmanuelle Charpentier and Jennifer A. Doudna for their outstanding discovery of CRISPR, which tremendously boosts this field. Following that, a number of studies have been carried out to explore the applications of CRISPR‐Cas systems. Among different classes of Cas proteins, class 2 systems, such as Cas9, and their derived variants, are one of the most studied, which encompass a single multidomain nuclease combining all activities that are required for gene interference.^[^ ^298^ ^]^ Once Cas9 is combined with the single guided RNAs (sgRNA)s, a Cas9 ribonucleoprotein complex will be formed, which triggers the double‐stranded DNA (dsDNA) break, activating dsDNA break repair machinery. The sgRNAs can be designed to bind to the designated location of the disease genes, thus allowing for specific gene targeting and manipulation. Indeed, by manipulating a library of genes in the cell models of brain diseases, they are being used to discover the pathogenic genes and develop potential therapies for CNS diseases.

There are mainly three approaches to CRISPR‐Cas9 application to treat CNS diseases. Among them, the most commonly adopted approach is to disrupt the expression of disease‐associated genes by creating DSBs and indel, which is a random insertion of the gene of interest after the DSBs are created. This approach is useful in treating CNS diseases when the causative genes or genetic risk factors are still under exploration, or the causative genes are too short to be cut. For example, CRISPR‐Cas9 was used to validate the effects of potential disease‐related genes and disease pathogenesis. In a study by Alcalay et al., they designed two guide RNAs and applied CRISPR‐Cas9 to knockout a PD‐associated gene *SMPD1* which encodes lysosomal enzyme acid‐sphingomyelinase (ASMase) in HeLa cells. They found such treatment resulted in decrease of ASMase activities, and potentially attributed to *α*‐Syn protein accumulation, thus supporting an association between *SMPD1*, ASMase activity, and PD.^[^ ^299^ ^]^ Park et al. intracranially injected the CRISPR‐Cas9‐loaded nanocomplex, targeting BACE‐1, into the brain of 5XFAD transgenic mice, an AD disease model. They showed that the nanocomplex was able to create indels to disable the BACE‐1 gene in the disease mice as evidenced by significant reduction of APP in the mouse brain.^[^ ^95^ ^]^ A less invasive approach was also explored by intravenous administration of CRISPR/Cas9‐loaded liposome‐templated hydrogel NPs, targeting polo‐like kinase 1 (PLK1), in both orthotopic and subcutaneous GBM mouse tumor models. Significant reduction in tumor volume was observed in these two models.^[^ ^32^ ^]^ Besides, the targeting specificity could also be increased by making use of the alleles of CNS diseases. For instance, György et al. used CRISPR‐Cas9 to specifically target APPswe (Swedish) mutation *APP* gene of AD patient‐derived fibroblast. They designed a sgRNA that can specifically recognize mutant *APP* (*mAPP*) gene based on the single nucleotide polymorphism (SNP) of the APPswe mutation. Sanger sequencing showed that the CRISPR‐Cas complex specifically cut the pathogenic gene without disrupting the wild type *APP* gene.^[^ ^93^ ^]^ CRISPR‐Cas in stroke treatment studies has been mostly limited to prevention instead of post‐treatment at this stage. For example, Zhou et al. first disrupted the expression of PlexinB1 gene by intracranial injection of the CRISPR‐Cas9‐containing lentivirus in rats 5 days before the generation of the stroke disease model. By means of MRI, they showed that such an approach reduced the BBB damage after stroke.^[^ ^300^ ^]^

Instead of disrupting the expression, correcting the gene of interest is the second approach of CRISPR‐Cas application in CNS disease area. Since the pathogenic gene of HD, mainly the CAG expansion in *HTT* gene, is well established, and this gene is long enough to be edited, HD treatment is a good example for demonstrating gene correction via CRISPR‐Cas. HD can be treated by removing the disease gene by cutting both ends simultaneously. At this stage, viral carriers remain the main strategies for delivering CRISPR‐Cas9 complex, and non‐viral delivery systems for this approach are still scarce. For example, Yang et al. first designed 2 guide RNAs (gRNAs) to target both sides of HTT, and they found that the transfection of HEK293 cells with Cas9 and these two gRNAs using lipofectamine significantly reduced the expression of HTT. Following this, they injected two AAV vectors expressing two gRNAs and Cas9 respectively (1:4) to the striatum of HD mice (HD140Q‐K1), which dramatically decreased the nuclear accumulation and aggregation of mHTT in the mice brain. Behavioral tests also showed that these mice had alleviated motor deficits and neurological symptoms, which further suggested that CRSPR‐Cas9 could be applied to effectively deplete *HTT* expression and permanently eliminate polyQ expansion‐mediated neuronal toxicity in the adult brain.^[^ ^301^ ^]^ However, this method reduces both wild type and mutant HTT proteins. Note that complete suppression of wild type HTT may result in shortened life span.^[^ ^302^, ^303^ ^]^ Given this, attempts have also been made to target the *mHTT* in the specific allele of HD. Monteys et al. intrastriatally injected two AAV vectors to the right striatum of mice carrying the full length of *mHTT* gene. One vector carrying the CRISPR/Cas9 complexes used the single nucleotide polymorphisims (SNP)‐dependent PAMs recognizing the SNPs at the upstream of HTT exon‐1. The other one used the common PAM to target HTT intron‐1. Cutting both upstream and downstream ends of *mHTT* exon‐1, the mHTT expression was decreased in the right hemisphere while the expression in the left was unchanged.^[^ ^304^ ^]^ Besides, the mutant forms of Cas9, such as Cas9 nickase (Cas9n), known to be safer and more specific than wild‐type Cas9, have also been explored. Dabrowska et al. electroporated patient‐derived HD fibroblasts with plasmids containing sgRNAs‐Cas9 nickase (Cas9n), which precisely excised the CAG repeats from the *HTT* gene and decreased HTT protein production.^[^ ^305^ ^]^ However, the authors somehow did not test such an effect on any HD animal model, which needs to be further confirmed.

The third approach other than performing irreversible gene editing, is to modulate the gene expression reversibly by disabling the cleavage function of Cas9 and adding an additional component to the Cas9. The disabled Cas9 is called dead Cas9 nuclease (dCas9). Losing the ability to cleave, dCas9 can still recognize the target gene and perform other functions if dCas9 is fused with addition domains. For example, Heman‐Ackah et al. fused Krüppel associated box (KRAB), a transcription repressor, to the dCas9, which reversibly suppressed PD‐related gene *SNCA* expression in induced pluripotent stem cells (iPSC)‐derived neurons.^[^ ^306^ ^]^ Besides, promoting neuroprotective gene expression is an alternative strategy for treating brain diseases. For instance, Chen et al. developed a screening platform called PRISM (Peturbing Regulatory Interactions by Synthetic Modulators) that uses CRISPR‐Cas9‐based transcription factors (crisprTFs). By using this platform, they identified a strong protective gRNA that could specifically promote the expression of protector genes, including DJ‐1, TXN, and TIMM9, leading to a significant decrease of *α*‐Syn toxicity in a yeast model of PD.^[^ ^307^ ^]^ Other than these three approaches, many different approaches remain to be explored in the context of CNS diseases such as RNA‐targeting Cas RNP and Cas9 variants for single‐nucleotide editing.^[^ ^308^ ^]^

In short, the CRISPR‐Cas9 system provides a new possibility of precisely exploring or manipulating CNS disease genes by either stopping, correcting, or reversibly altering the expression of the gene of interest. Despite many promising results, the CRISPR‐Cas9 approach still has not entered clinical trials for CNS diseases.^[^ ^309^ ^]^ CRISPR‐Cas9 for in vivo application is typically delivered via viral vectors (e.g., AAV). This is a relatively mature technique in the field but suffers from issues like immunogenicity, limited transgene capacity (around 4.8 kb), and is hard to stop once the cells start to express the CRISPR‐Cas9 RNPs.^[^ ^310^ ^]^ To make gene editing more controllable, direct injection of CRISPR‐Cas9 to the brain has been shown to exhibit targeting activities in the brain like editing post‐mitotic neurons in brain, inhibiting the growth of GBM.^[^ ^311^ ^]^ However, this is too invasive for clinical translation. Therefore, there is a need to develop non‐viral delivery approaches, especially nano‐carriers, of the CRISPR‐Cas9 complex to reduce the invasiveness of the administration route, target the disease sites, and protect the large Cas9 protein and highly negative sgRNA during the delivery.^[^ ^312^ ^]^ Nanocarriers such as gold NPs,^[^ ^31^ ^]^ magnetic NPs^[^ ^313^ ^]^ are promising delivery approaches for less‐invasive and targeted delivery of CRISPR‐Cas9 system to CNS disease sites and may overcome some of the hurdles of bringing CRISPR‐Cas9 into the clinic.

### 3.7 Backbone Modification

**Figure 3:**
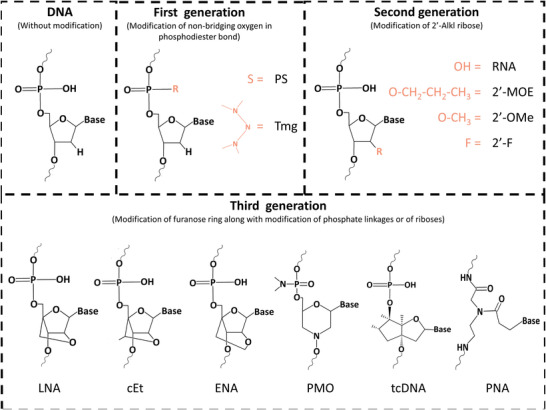
Backbone modifications. There are many different backbone modifications for the oligonucleotides that can be classified from the first generation to the third generation.^[^ ^321^, ^322^ ^]^ Examples of the modifications are listed above: DNA, deoxyribonucleases, PS, phosphorothioate; Tmg, internucleotide phosphate modified with a tetramethyl phosphoryl guanidine group; RNA, ribonucleases; 2′‐MOE, 2′‐O‐methoxyethyl (a methoxyethyl substitution in the 2′ position of the sugar moiety); 2′‐OMe, 2′‐O‐methyl (a methyl substitution in the 2′ position of the sugar moiety); 2′‐F, 2′‐fluoro substitution in the 2′ position of the sugar moiety; LNA, locked nucleic acid (an extra bridge between 2′ oxygen and 4′ carbon of the sugar moiety); cEt, constrained ethyl (an ethyl bridge between 2′ oxygen and 4′ carbon of the sugar moiety); PMO, phosphorodiamidate morpholino oligonucleotide, or morpholino (backbone composed of methylenemorpholine rings and phosphorodiamidate linkages); ENA, 2′‐O,4′‐C‐ethylene‐bridged nucleic acid (an ethylene bridged at the furanose sugar ring at 2′‐O and 4′‐C ends); tcDNA, tricyclo‐DNA (3 additional C‐atoms between C(5′) and C(3′) of the sugar moiety); PNA, peptide nucleic acid (entire sugar phosphate backbone is replaced with polyamide linkage).

Bare nucleic acids usually suffer from issues like in vivo degradation and fast clearance, therefore, various backbone modification strategies have been developed for modifying nucleic acids (e.g., siRNA, AON), such as phosphorothioate (PS), 2′‐O‐Methyl (2′‐O‐Me), 2′‐O‐Methoxyethyl (2′‐MOE), 2′Fluoro (2′‐F), locked nucleic acid (LNA) and morpholino (PMO). Several reviews have extensively summarized different backbone modifications.^[^ ^257^, ^314^, ^315^ ^]^ The focus here is on the most commonly used backbone modifications to enhance the stability, cellular uptake, and binding affinity of genetic modulators (**Figure** **3**). For instance, PS modification, the first generation of modification (non‐bridging oxygen atoms in the phosphate group), is commonly introduced to confer nucleic acids with nuclease resistance and prolong their circulation half‐life.^[^ ^316^ ^]^ PS modification also recruits intracellular proteins (e.g., nucleolin)^[^ ^317^ ^]^ that may be responsible for intranuclear transport of AON. It is also found that they can accumulate in a number of membrane‐less structures in the nucleus, nucleolus, and cytoplasm.^[^ ^318^ ^]^ Note that PS modification of AON was reported not to disrupt activities of RNase H, but PS modification on all the bases of siRNA resulted in reduced gene silencing ability.^[^ ^319^, ^320^ ^]^ Therefore, PS modification is usually not singly used, but combined with other modifications to optimize the interaction between AON and the RNase H.

Second‐generation backbone modifications include alkyl modification at 2′ position such as 2′‐O‐Me, 2′‐MOE, 2′‐F. Of these, 2′ribose modifications for siRNA (e.g., 2′O‐Me and 2′‐F) were shown to increase the loading of siRNA onto RISC, leading to increased gene silencing potency.^[^ ^323^ ^]^ More than 500‐fold increase in siRNA potency was observed in the studies conducted by Allerson et al.^[^ ^324^ ^]^ It is also found that 2′‐O‐Me can be incorporated into siRNA for reducing the associated immune response.^[^ ^234^ ^]^ However, these 2′ribose modifications do not support RNase H cleavage. Unless the intended application is to simply block the target gene, the gapmer design would be adopted. A gapmer usually contains 2 to 5 number of bases that have a second or third (see below) generation of modification at each terminus only, which allows the binding of RNase H to the flanking central “gap” of the AON.^[^ ^325^ ^]^ This design affords stronger binding to the target gene while maintaining the ability of AON to load onto RNase H. The first two generations of modifications are usually used together for specific CNS diseases. Indeed, several clinical trials are currently being carried out to study the modified AON in various CNS diseases. For instance, Ionis Pharmaceutics launched a phase 1b/2a clinical trial to treat HD. The intrathecally injected AON were PS and 2′‐MOE modified and the results showed a dose‐dependent reduction of pathogenic proteins (NCT02519036).^[^ ^184^ ^]^

To further increase the binding affinity, a third‐generation modification, mainly modifying the furanose ring such as PMO and LNA, was developed. These modified nucleic acids exhibited strong affinity to the target gene and do not recruit RNase H, which are the desired properties to block target genes.^[^ ^182^ ^]^ For instance, tricyclo‐DNA (tc‐DNA) was shown to have an effect on brain upon intravenous administration.^[^ ^326^ ^]^ Such modification incorporated into a gapmer also showed an increased therapeutic effect for HD treatment following the intracranial injection.^[^ ^182^ ^]^

## 4 Blood Brain Barrier

The BBB is an intact metabolic and physical barrier that regulates the homeostasis of the CNS. It is one of the most important interfaces for the molecules, such as ions, nutrients, and cells, between blood and neurons. The BBB is mainly formed by cerebral endothelial cells together with significant presence of pericytes, astrocytes, and neural cells interconnected by tight junctions.^[^ ^327^ ^]^ The tight junction of the BBB protects the brain from being harmed by unwanted and toxic substances and controls the CNS microenvironment, which is essential for normal neuronal functions. In many CNS diseases, including AD, PD, HD, and stroke, the integrity of the BBB is usually disrupted to some extent. In brain cancers, the disrupted BBB is also referred to as blood‐brain tumor barrier (BBTB). Despite being compromised under these disease conditions, the tight junctions of the BBB still restrict the entrance of the external molecules into the brain parenchyma, including nanomedicines carrying nucleic acids into the brain, thus limiting their gene transfection efficiency.

Delivery of most molecules, therapeutics, and NPs into the brain involves passing through a tight junction. Generally, the transport through the brain endothelial cells mainly includes two pathways: paracellular and transcellular pathways. Only certain types of cells (e.g., monocytes, macrophages, and neutrophils) and small molecules (e.g., glucose, gases, hydrophobic molecules less than 500 Da) could be passively diffused into the plasma membrane of the endothelial cells and enter the brain, which is called a paracellular pathway.^[^ ^328^ ^]^ For the majority of other substances, including NPs and most therapeutic agents, transcellular pathways play a main role in transportation into the brain. Three categories are mainly included in the transcellular pathways: carrier‐mediated transcytosis, receptor‐mediated transcytosis, and adsorptive‐mediated transcytosis. The efficiency of transporting NPs into the brain is largely influenced by the properties of NPs used, such as their size, charge, shape, morphology, and surface modification. Owing to the high efficiency and specificity towards the brain, the receptor‐mediated transcytosis is considered as one of the most promising approaches to facilitate NPs crossing the BBB, which is usually achieved by functionalizing the nanocarriers with the brain‐targeting ligands. These targeting ligands will drive NPs to bind to the surface receptors on the luminal side of brain endothelial cells, triggering endocytosis and vesicular trafficking processes that transport NPs to the abluminal side of brain endothelial cells.

Up to date, a wide range of surface receptors has been identified to be highly expressed in the brain endothelial cells, including transferrin receptor, low‐density lipoprotein receptor (LRP), and insulin receptor (IR). These receptors and their ligands are commonly utilized for brain‐targeted delivery. A number of other receptors, such as scavenger receptor, glutamate receptor, lactoferrin receptor, and folate receptor, are also being inclusively studied for brain targeting of various NPs (reviewed by Xie et al.).^[^ ^328^ ^]^

To allow for targeting the specific receptors, many targeting ligands have been applied to functionalize the NPs, including the natural ligands such as transferrin, insulin, leptin, Apo‐A and E, receptor‐related protein, glucose, p‐hydroxybenzoic acid. For example, transferrin, which targets transferrin receptors on brain endothelial cells, has been widely used to conjugate to various NPs (e.g., AuNPs, pSiNPs, exosomes, lipid‐based NPs), and significantly improved their BBB penetration ability. Indeed, most of the targeted NPs in the clinical trials target transferrin/transferrin receptors.^[^ ^329^ ^]^ However, it should also be noted that the natural ligands may compete with their endogenous counterparts. A typical example is that the transferrin receptor is usually saturated when the concentration of endogenous transferrin is over 25 um in the blood.^[^ ^330^ ^]^ Therefore, a higher concentration of NPs will be required to compete with endogenous transferrin. To avoid such competition, lactoferrin (Lf), a transferrin family member, which has a slower concentration in the plasma (5 nm), has also been used to functionalize NPs, and facilitate their BBB penetration.^[^ ^331^ ^]^

Antibodies with high specificity have also been broadly applied to functionalize NPs to target the surface receptors on brain endothelial cells. They could be developed to target the domain of the receptor that is not used by the endogenous ligands, therefore avoiding the competition with endogenous counterparts in the body.^[^ ^332^ ^]^ The efficiency of BBB penetration could be influenced by the affinity and the density of antibodies on NPs. Generally, BBB penetration will benefit from a high density of antibodies on the NPs. Interestingly, the brain uptake of NPs may be compromized by a high affinity of antibody, while benefiting from a lower affinity as found in several studies.^[^ ^333^, ^334^ ^]^ Most NPs are modified with the full‐length antibodies produced from non‐human species, such as murine and rats. Since non‐human‐originated antibodies are normally immunogenic in humans and have poor interaction with human cells, some antibody derivatives are also being developed, such as the combinatory antibodies targeting the receptors with human constant regions.^[^ ^335^ ^]^ These humanized antibodies usually contain complementary‐determining regions (CDRs) within variable regions from non‐human origin (such as murine) and the constant regions from human origin.^[^ ^336^ ^]^ They have decreased immunogenicity while still maintaining a high affinity with human cells. Another attractive derivative is the single chain Fv fragment that only contains the variable domains of the heavy and light chains. Compared to the full‐length antibodies, these fragments have a smaller size and could be produced at a lower cost owing to their simple structure while still having a high targeting ability.^[^ ^337^ ^]^ However, it should be noted that these scFv fragments do not contain the Fc regions, so they have relatively shorter half‐lives in the blood circulation.^[^ ^338^ ^]^ This could be potentially improved by further engineering, such as adding a cysteine to scFV to form a bisulfide bond between to scFvs.^[^ ^335^ ^]^ Though these antibodies have not been used to modify NPs for BBB penetration purposes so far, they offer a potential application in this field in the future.

Peptides are another commonly studied ligands that are used for NP functionalization to assist BBB crossing. Compared to antibodies, they are easily synthesized at a lower cost without compromising the high specificity. Peptides can be used to improve the passive transportation at the BBB including those with high lipophilicity and the ability to interact with the hydrogen bonds at the plasma membrane, such as diketopiperazines (DKPs), N‐MePhe, and (PhPro)_4_ family.^[^ ^339^ ^]^ However, these small peptides are usually more appropriate for conjugation to small molecules. For NPs, peptides that facilitate active brain transportation are more adequate. CPPs, which are molecules mainly have amphipathic or cationic sequences, can facilitate the brain entry of NP mainly via the interaction with the plasma membrane.^[^ ^340^ ^]^ Several CPP candidates, such as TAT and R8, have been used as brain‐targeted ligands.^[^ ^341^, ^342^ ^]^ However, it should be noted that CPP‐mediated brain entry is usually not specific to certain receptors. Most of the studies focus on developing the peptides targeting the specific receptors within the BBB and facilitating the active transportation, such as, T7 targeting TfR,^[^ ^343^ ^]^ Angiopep‐2 targeting LDR‐1,^[^ ^344^ ^]^ and ApoE‐derived peptides targeting LDL receptors.^[^ ^345^, ^346^ ^]^ RVG, a peptide derived from the rabit virus glycoprotein, targeting nicotinic acetylcholine receptor (nAchR), is one of the most extensively used peptides to modify various NPs for BBB penetration.^[^ ^347^, ^348^ ^]^ Besides, various strategies are being investigated to identify other potential peptides that could facilitate BBB transportation with high specificity, such as phage display, computer‐based techniques, and chemical libraries screening.^[^ ^349^, ^350^ ^]^ A number of BBB shuttle peptide families have been newly discovered via these approaches (summarized in the review by Macarena et al.),^[^ ^339^ ^]^ which provide great potentials to guide NPs to enter the brain.

In addition, numerous other ligands for NPs modification are also broadly investigated, such as cationic bovine serum albumin (CBSA), and Opca protein of *Neisseria meningitidis*.^[^ ^351^ ^]^ Nucleic acids, which target scavenger receptors at the BBB, are emerging as brain‐targeted ligands leading to enhanced BBB penetration.^[^ ^352^ ^]^ Some other strategies have also been explored to facilitate the transportation of nanomedicines across the BBB. Temporary opening of the BBB physically such as focus ultrasound, or by chemicals (e.g., mannitol) have been demonstrated to enhance BBB penetration of NPs in both animal studies and clinical trials.

## 5 Nanomedicines Used in Gene Delivery for CNS Disease Treatment

### 5.1 Inorganic NPs

Inorganic NPs have special physicochemical, optical, and electrical features. Compared to the organic NPs, inorganic NPs have a solid structural backbone which could provide strong physical support for the cargo inside.^[^ ^353^ ^]^ It is common to modify the surface of inorganic NPs with biocompatible materials such as targeting ligands, biomolecules, stabilizers (e.g., polymers, dendrimers, surfactants), to enhance their colloidal stability, BBB penetration, capacity in delivering genetic agents, as well as specificity to the diseased sites within the brain. To date, a variety of inorganic NPs has been developed for such applications, including gold NPs, magnetic NPs, quantum dots (QDs), silica/silicon NPs, and copper NPs, which will be detailed in the following sessions.

#### 5.1.1 Gold NPs

Gold NPs (AuNPs) are one of the most common inorganic nanomedicines used in biomedical applications, owing to their advantages in easily tunable sizes and shapes, high affinity for biomolecules, and easy and cost‐efficient synthesis/modification. Interestingly, a study by Kaikai et al. shows that bare AuNPs could abundantly activate the neuron‐specific genes (e.g., *SYN*, *MAP2*, *TH*, and *DAT*) and thus increase the dopamine level of PD mice under the presence of electromagnetic field.^[^ ^354^ ^]^ In the mid‐2000s, Mirkin and colleagues covalently conjugated AON to AuNPs, which yielded a tremendously improved cellular uptake in the brain through scavenger receptors and an enhanced gene knockdown efficiency.^[^ ^355^, ^356^ ^]^ Consequently, AuNPs have been widely used to conjugate with nucleic acids for CNS disease theranostics. Mirkin's group developed a GBM‐targeted spherical nucleic acid (SNC) by covalently functionalizing AuNPs with siRNA, which have been demonstrated to transverse the BBB and modulate the target oncogene *BCL2L12* in the in vitro and in vivo GBM models.^[^ ^352^ ^]^ Additional to directly targeting *BCL12L12*, miRNA‐conjugated AuNPs targeting miR‐182 were also fabricated, which further diminished the expression of several miR‐182‐regulated oncogenes, including *BCL2L12*, *c‐Met*, and *HIF2A*. The conjugate was found to reduce the tumor burden and improve the survival in orthotopic GBM animal models after intravenous administration.^[^ ^357^ ^]^ Following the promising preclinical results, an early phase clinical study (NCT03020017) is currently ongoing to evaluate the safety of NU‐0129 (siRNA‐coated AuNPs targeting *BCL2L12*) in GBM patients after intravenous injection, which is one of the few nanomedicines‐based gene therapies for CNS diseases in clinical trials. Notably, to obtain therapeutic benefits, oligos should be released from the NPs intracellularly upon exposure to the specific stimulus. This could be achieved by involving a cleavable linker between nucleic acids and AuNPs. For example, a disulfide bond, which is stable in the extracellular environment, but is cleaved by the intracellular glutathione (GSH), is commonly used to conjugate nucleic acids to AuNPs as exampled by Cheng et al.^[^ ^358^ ^]^

**Figure 4:**
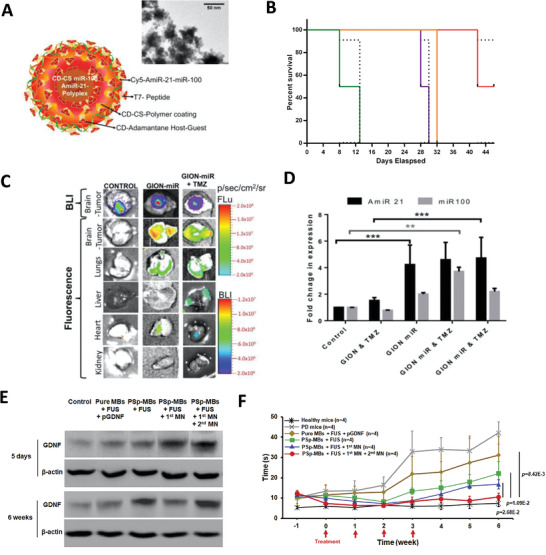
Inorganic NPs for gene therapy in CNS diseases. A–D) Gold‐iron oxide NPs (GION) for miRNA delivery for GBM treatment. (A) Schematic representation of GION structures and representative TEM image. GION are coated with *β*‐cyclodextrin‐chitosan (CD‐CS) hybrid polymer and loaded with miRNA (miR‐100 and antimir‐21), and modified with targeting ligand PEG‐T7. (B) The survival curve of GBM mice after intranasal administration of control (green), GION&miR (purple), TMZ (orange), and GION miR&TMZ (pink). (C) ex vivo fluorescence images and (D) RT‐PCR quantification of miRNA in various organs of mice treated with GION‐CD‐CS‐miRNA‐T7. (A–D) Reproduced with permission.^[44]^ Copyright 2019, Elsevier Ltd. E,F) Superparamagnetic iron oxide microtubes for plasmid DNA delivery for PD. E) The *GDNF* expression measured by Western blot analysis. The animals were sacrificed after 5 days and 6 weeks of treatment. F) Motor ability of PD mice measured by the beam‐walking test. Times to cross 80 cm beam were recorded. (E,F) Reproduced under the terms of the Creative Commons CC BY NC ND license.^[^ ^362^ ^]^ Copyright 2020, The Author(s), published by Elsevier Inc.

Besides directly conjugating nucleic acids to AuNPs, they are also often physically absorbed onto the AuNPs‐based nano‐systems via electrostatic interactions. To enhance the adsorption efficiency, AuNPs have been modified with various molecules, such as dendrimers,^[^ ^359^ ^]^ polyethylene glycol (PEG), transferrin (Tf),^[^ ^360^ ^]^ polyetherimide (PEI),^[^ ^361^ ^]^ and NGF.^[^ ^121^ ^]^ For example, in a study by Sukumar et al., gold‐iron oxide NPs were functionalized with PEG‐T7, which led NPs to cross the BBB and reach the GBM tumor site upon the exposure through intranasal inhalation. The cocktail cargos delivered by this system, including the genetic modulators (antimiR‐21‐miR‐100) and chemo drug TMZ, sufficiently increased the survival rate of a GBM orthotopic mice model (**Figure** **4**A–D).^[^ ^44^ ^]^ Another study by Hu et al. reported that plasmid DNAs were loaded into chitosan/NGF‐modified AuNPs for PD treatment. The resulted nanocomplexes successfully decreased the expression of *SNCA* gene and displayed an efficient therapeutic effect in both in vitro and in vivo PD models.^[^ ^121^ ^]^ Compared to GBM and PD, the applications of AuNPs for gene therapy development in AD, HD, and stroke are relatively rare.

Owing to the unique optical properties, AuNPs have been used as photothermal agents in various techniques for CNS disease diagnosis and therapies, including sensing, labeling, and imaging.^[^ ^363^, ^364^ ^]^ Indeed, AuNPs have been widely used in biosensor development for disease‐implicating genes detection in AD^[^ ^365^, ^366^ ^]^ and PD.^[^ ^367^ ^]^ For example, Delkhahi et al. developed an enzyme‐free biosensor for the detection and quantification of miR‐137 by combining AuNPs‐based colorimetric detections and DNA hybridization chain reaction (HCR) amplification. This biodevice allowed for a high sensitivity within the linear range of 0.25–5 nm.^[^ ^366^ ^]^ In recent work, miRNA detection was achieved for PD by coating AuNPs with black hole quencher 2 (BHQ2) labeling hairpin DNA (AuNP@hDNA‐BHQ2), which showed a quick (35 min) and highly sensitive (fM level) response to miR‐5010 and miR‐331.^[^ ^367^ ^]^

Notably, the properties of AuNPs, including cellular internalization, biodistribution, gene delivery efficiency, and optical properties, may change dramatically when the size and/or the shape of spherical AuNPs change. This should be taken into consideration before using AuNPs to deliver gene modulators. Various comparison studies have been carried out. An interesting work done by Jun et al. reported the influence of the sizes and shapes of the AuNPs on the cellular uptake and intracellular distribution in a GBM cell model.^[^ ^368^ ^]^ In this study, 50‐nm spheres and 40‐nm stars showed higher gene delivery efficiency compared to 13‐nm spheres. Among various shapes, gold nanospheres and nanorods^[^ ^359^ ^]^ are the most commonly used for gene delivery in CNS disease studies. Besides, nanostars, nanoshells, nanoclusters, and nanoflowers have also been reported for gene delivery in non‐CNS disease studies (reviewed by Artiga).^[^ ^369^ ^]^

#### 5.1.2 Magnetic NPs

**Figure 5:**
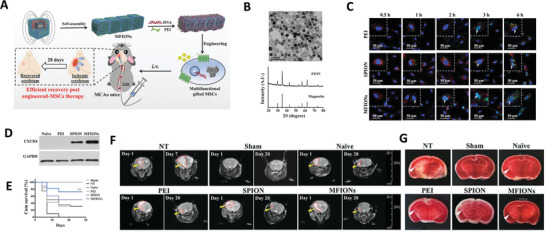
Magnetic NPs to combine gene delivery and cellular therapy for stroke. A) Schematic illustration of MSCs therapy for post‐ischemic stroke based on magnetosome‐like ferrimagnetic iron oxide nanochains (MFIONs). B) TEM image of ferrimagnetic iron oxide nanocubes (FIONs) used to assemble MFIONs (Top). Powder XRD patterns of the synthesized FIONs compared with the standard Fe_3_O_4_ (Bottom). C) Confocal images of the process of cellular internalization, the subsequent endolysosomal escape, and nuclear import in the delivery of FITC‐DNA by indicated vectors. Blue: nucleus. Green: FITC‐DNA; Red: endolysosomes. White arrows indicate the nuclear import of FITC‐DNA. D) CXCR4 expression in MSCs 24 h after given treatments. E) Survival curves of ischemic mice with the given treatments. F) Reduction of infarct volume examined by MRI (indicated by red dash lines and yellow arrows), and G) TTC staining (indicated by white arrows). It suggested a therapeutic effect of the engineered‐MSC by magnetic NPs in the recovery post‐ischemic stroke. Reproduced with permission.^[^ ^379^ ^]^ Copyright 2019, WILEY‐VCH.

Magnetic NPs (MNPs) as gene delivery systems for CNS diseases have been widely investigated owing to their unique superparamagnetic feature, which allows the guided and targeted gene delivery under an external magnetic field.^[^ ^370^ ^]^ Typically, an MNP consists of a magnetic core (e.g., iron, nickel, cobalt, and their oxides) and a non‐magnetic coating via various surface chemistry (e.g., NGF, PEG, TWEAK), which enables nucleic acid delivery and tissue/cell penetration.^[^ ^371^, ^372^, ^373^ ^]^ Among these, iron oxide NPs (Fe_3_O_4_, or its oxidized and more stable form Fe_2_O_3_) are the most commonly explored for biomedical applications due to their biocompatibility and biodegradability.^[^ ^374^ ^]^ With an external magnetic field, the MNPs carrying nucleic acids can be guided to adhere to the cell membrane, thus increasing their accumulation in the target cells. Such magnetism could be simply terminated by removing the magnetic field resource. Although the MNP‐induced cellular accumulation could not impact the nuclear targeting and internalization of genetic materials, the increased accumulation of genes in the cytoplasm potentially facilitates nuclear translocation and infiltration.^[^ ^375^ ^]^ The unique feature has attracted exclusive attention in various theragnostic applications in CNS diseases, including targeted gene delivery for PD,^[^ ^371^ ^]^ GBM,^[^ ^373^, ^376^ ^]^ AD,^[^ ^377^ ^]^ and stroke.^[^ ^378^ ^]^ A typical example was done by Wu et al., who applied PEI‐modified MNPs to deliver a plasmid DNA encoding GDNF to PD mice. With the assistance of magnetic navigation, these plasmid DNA displayed enhanced local concentration and intracellular trafficking into nucleus in the brain of testing animals, which showed a significant increase in dopaminergic neuron recovery and motor behavior (Figure 4E,F). It is noted that the authors first opened the BBB by using focused ultrasound (FUS) before the treatment.^[^ ^362^ ^]^ Another study by Zhang and colleagues developed magnetosome‐like ferrimagnetic iron oxide nanochains (PEI‐MFIONs) to deliver the *BDNF*‐targeted plasmid DNA for schematic stroke treatment (**Figure** **5**).^[^ ^379^ ^]^ They found that the external magnetic field increased the gene transfection efficiency of superparamagnetic iron oxide NPs (SPION) as expected, whilst it only had a limited influence on that of MFIONs, which was plausibly achieved by the chain‐like structure of MFIONS with the increased aspect ratios. Notably, this is the first study that transplanted genetically modified mesenchymal stem cells (MSCs) into ischemic stroke mice via intravenous injection. This approach led to increased neuroprotection and quick cerebral recovery. Such an external magnetic field‐independent system may address the issues of the large distance between the external magnetic field and the pathological site, which could tremendously increase the flexibility of MNPs for clinical applications.

Additionally, as an ideal contrast agent, MNPs could be directly visualized and tracked under the magnetic resonance imaging system (MRI), which allows for guided gene delivery. This unique feature was used in MRI‐guided theranostics and tumor imaging for CNS gene delivery as reported in several studies.^[^ ^44^, ^378^, ^380^, ^381^ ^]^ Nevertheless, the practical application of MNPs in gene delivery to CNS disease patients is still at an early stage owing to several challenges. The safety concerns of MNPs remain the primary obstacle that hinders the further application of MNPs. Though iron oxide is generally considered safe, it is known that imbalanced homeostasis can cause a toxic impact on many organs, including the brain.^[^ ^382^ ^]^ As the abundant redox‐active metal, iron could result in the production of free radicals in the brain, and excess iron has been identified to be associated with some CNS diseases accompanied by cellular damage and oxidative stress, including AD,^[^ ^383^ ^]^ PD,^[^ ^384^ ^]^ HD,^[^ ^385^, ^386^ ^]^ and stroke.^[^ ^387^ ^]^ Therefore, studies have to be carried out to explore the neurotoxicity of MNPs and identify the safe formulations prior to their therapeutic use for CNS diseases. Another barrier of MNPs is the poor uniformity and large batch‐variations during large‐scale synthesis, which lead to negative impact on the physicochemical properties of MNPs and thus compromise their gene delivery efficiency in the practical applications.^[^ ^375^ ^]^

#### 5.1.3 Quantum Dots

QDs, which are usually colloidal semiconducting nanomaterials, have become a recent focus for gene delivery owing to their unique optical and electronic properties.^[^ ^388^, ^389^ ^]^ Historically, many fluorescent NPs, such as silica, germanium, gold, silver, carbon dots, and graphene dots, have been referred to QDs.^[^ ^390^, ^391^, ^392^, ^393^ ^]^ In a related review, Niko et al. narrowed the definition of QDs to those prepared with core, core‐shell, or core‐multishell configurations from binary, tertiary, alloyed, or other semiconductors in nanocrystalline form (e.g., CdSe, ZnS).^[^ ^388^ ^]^ QDs have tunable size and versatile surface chemistry which allow conjugating with nucleic acids and other biomolecules (e.g., proteins and antibodies). They have been used to deliver and/or detect the genetic modulators for CNS diseases. A typical example was done by Lin and colleagues, who modified CdSSe/ZnS QDs with PEI and subsequently embedded *TERT*‐targeted siRNA by electrostatic interaction.^[^ ^394^ ^]^ The as‐designed nano complexes were achieved to silence the *TERT* gene by around 50% at gene expression and protein level in two GBM cell lines. Another work done by Perini showed that bare graphene QDs with various surface terminals such as amine and carboxyl were generally biocompatible and did not affect DNA integrity. They induced 20% cytotoxicity on GBM cells when the concentration went above 250 µg mL^−1^, and showed a synergetic effect with TMZ on GBM cells but not on neurons.^[^ ^395^ ^]^ Notably, certain types of QDs, such as graphene QDs, have been reported to possess the capability to cross biological barriers, including the BBB.^[^ ^391^, ^392^ ^]^

**Figure 6:**
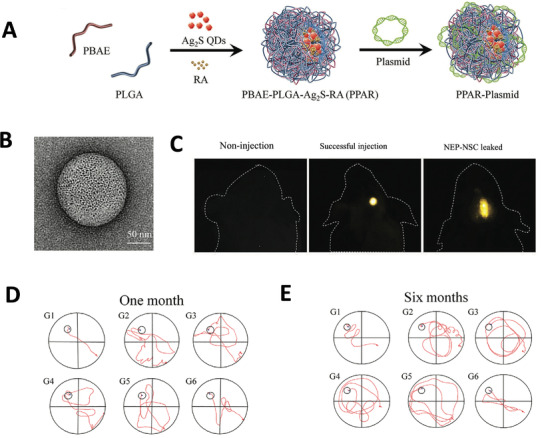
QD‐based gene therapy for CNS diseases. Ag_2_S QD‐based nanocomplexes for delivery of plasmid expressing neprilysin (NEP) for AD. The Ag2S fluorescence imaging is used to guide the real‐time transplantation of NSC. A) Synthesis of Ag_2_S QDs covered with polymer PBAE and PLGA (PPAR). Plasmid and RA were loaded into the nanocomplexes. B) TEM image of the PPAR nanocomplexes. C) In vivo NIR‐II imaging of the stereotactically transplanted NSCs using Ag_2_S QDs. D) The escape track assay during spatial learning and memory training of AD mice 1 month (D) and E) 6 month after treatment. G1: healthy mice; G2: PBS‐treated AD mice. G3: NSC‐treated AD mice; G4: nanocomplexes‐NSC‐treated AD mice; G5: NEP‐NSC‐treated AD mice; G6: nanocomplexes‐NEP‐NSC‐treated AD mice. Reproduced with permission.^[^ ^398^ ^]^ Copyright 2021, Wiley‐VCH.

QDs are considered a superior material over many fluorophores as they have desirable narrow spectra, bright photoluminescence, high quantum yields, broad optical absorption, and high photo‐ and chemical‐ stability. These unique optical properties allow us to visualize the internalization of NPs in the cell or animals. For example, the PEI‐modified carbon dots developed by Gui et al. yielded a strong red fluorescence and allowed to image the NPs inside the cells, which could directly track the siRNA delivery to GBM cells.^[^ ^396^ ^]^ In addition, QDs are often embedded into other nanoformulations to guide gene delivery. For instance, in a work by Yoon et al., QDs with different wavelengths, which served as the fluorescence labels, were photochemically immobilized into hydrogel microparticles for AD‐miRNA detection.^[^ ^397^ ^]^ A recent work by Dehua and colleagues encapsulated Ag_2_S QDs, plasmid DNA, and drug retinoic acid (RA) into their PLGA‐based nanocomplexes, which were applied to treat NSC. Ag_2_S QD‐based fluorescence imaging was used to guide stereotactic transplantation of NSC, thus achieving an accurate gene and drug delivery to hippocampus in AD mice.^[^ ^398^ ^]^ This strategy yielded improved A*β* clearance and neural regeneration in the testing animals (**Figure** **6**).

#### 5.1.4 Silicon‐based NPs

Silicon‐based NPs (SiNPs) are emerging as another attractive material being explored to deliver genetic materials for CNS diseases. There are two main types of SiNPs, elemental silicon NPs, and oxidized silica (SiO_2_) NPs, which are synthesized by different methods (typically exampled by Ermei^[^ ^399^ ^]^ and Meng,^[^ ^400^ ^]^ respectively). Silica and silicon NPs share many unique properties, including tunable particle size, desirable biocompatibility, and in vivo biodegradability, and versatile surface modification.^[^ ^399^, ^401^ ^]^ Up to date, many attempts have been made to engineer both types of SiNPs for CNS gene therapy, especially their porous variants, usually termed as mesoporous silica NPs (MSNs) and porous silicon NP (pSiNPs), respectively. In 2007, Klejbor and colleagues for the first time reported to apply MSNs as non‐viral gene delivery vectors for HD treatment. Following that, many independent groups employed MSNs to deliver siRNA, miRNA, and AON for brain diseases.^[^ ^390^, ^402^, ^403^ ^]^ In a work by Cheng et al., a nuclear plasmid expressing GTPases was absorbed onto MSNs.^[^ ^404^ ^]^ These plasmids on the surface not only functioned as genetic therapeutics but also as gatekeeper for the co‐loaded drugs. A neuro cell line N2a showed upregulated gene expression and neurite outgrowth after treatment with this nanocarrier. Our group also attempted to load siRNA into PEI‐modified pSiNPs, which successfully silenced the expression of *MRP1* encoding multidrug resistance‐associated protein and inhibited GBM growth both in vitro and in vivo.^[^ ^405^, ^406^, ^407^ ^]^ Notably, pSiNPs with large pores could be used to accommodate other small nanocarriers such as AuNPs, which are strategized as “NPs‐loaded NPs (NLN)”. As exampled by Jinhwan et. al., AuNPs were loaded into MSNs, which protected AuNPs in the blood circulation and allowed them to penetrate deeply into 3D tumors with anticancer drugs.^[^ ^408^ ^]^ Although this concept has not been used for CNS gene delivery so far, it provides a potential design strategy to transport genetic modulators together with small nanocarriers to achieve combined benefits, for example, MNPs for imaging, and AuNPs for therapy.

#### 5.1.5 Other Inorganic NPs

In addition to the abovementioned inorganic particles, other inorganic NPs such as copper NPs have also been used in CNS gene delivery. For example, Cui et al. reported that a copper NP‐based biosensor could detect miRNA as low as 1 fm within a short time (<2h).^[^ ^409^ ^]^ Despite the promising results in preclinical studies, the inorganic NPs have not been approved for gene delivery for brain diseases yet. Only a rare number of inorganic NPs have entered clinical trials so far. Several key challenges remain to be further solved, including their potential toxicity especially neurotoxicity, off‐target issues, agglomeration, and difficulty of uniform batch production.

### 5.2 Polymeric NPs

**Figure 7:**
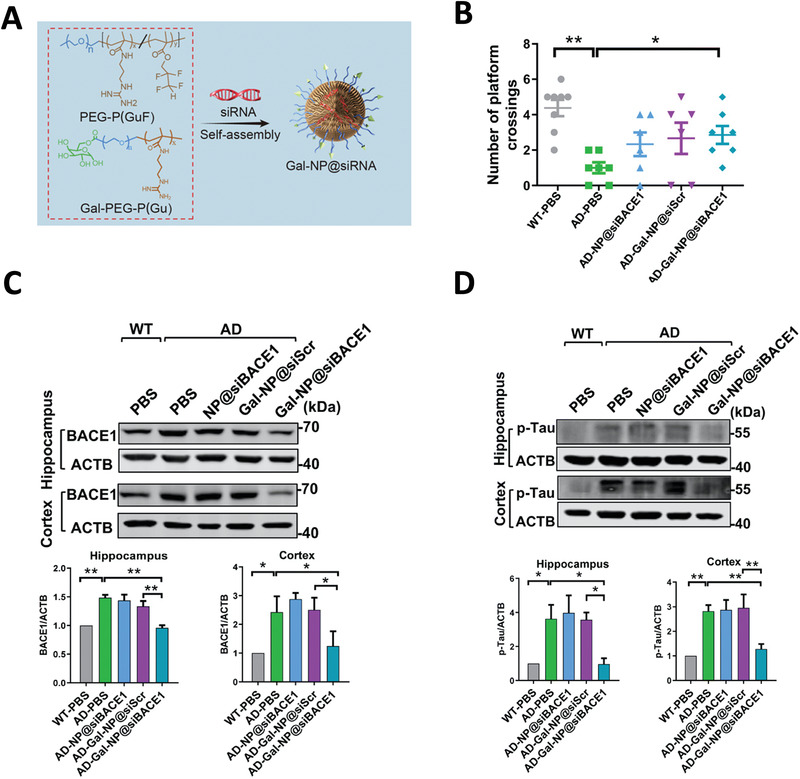
Engineered polymeric NPs for gene delivery for AD treatment. A) Schematic illustration of the formation of the glycosylated “triple‐interaction” stabilized siRNA NPs. B) Increased behavioral evaluation of siRNA‐carried polymeric NP‐treated APP/PS1 mice, by showing decreased number of crossing the platform location of each group on the probe test day. C) Decreased BACE1 protein expression in hippocampus and cortex from nanocarrier‐treated APP/PS1 mice. D) Reduced p‐tau expression in hippocampus and cortex from nanocarrier‐treated APP/PS1 mice. Reproduced with permission.^[^ ^53^ ^]^ Copyright 2020, The Authors, some rights reserved, exclusive licensee AAAS. Distributed under a Creative Commons Attribution NonCommercial License 4.0 (CC BY‐NC).

Polymer‐based nanoparticles (PNPs) have been emerging as one of the most extensively investigated gene delivery systems for CNS drug development. PNPs provide many advantages, including controlled and sustained delivery with desirable biocompatibility and biodegradability.^[^ ^410^ ^]^ Three types of PNPs are commonly used for brain gene delivery, including synthetic PNPs, natural‐based PNPs, and hybrid NPs.^[^ ^411^ ^]^ Several synthetic polymers are commonly used to fabricate PNPs, such as poly(lactic‐co‐glycolic acid) (PLGA), polyethyleneimine(PEI), polyethylene glycol (PEG), poly‐L‐lysine (PLL), poly(alkyl cyanoacrylates)s (PACA), poly(lactic acid (PLA), poly(caprolactone (PCL), and dendrimers.^[^ ^410^ ^]^ For example, Zhou and co‐workers used the PEG‐based PNPs to deliver siRNA targeting *BACE1* to the brains of APP/PS1 transgenic AD mice.^[^ ^53^ ^]^ In this study, PEG was modified with galactose to facilitate the BBB penetration of NPs (**Figure** **7**A). Interestingly, instead of relying on a sole electrostatic reaction, the authors employed a “triple‐interaction” stabilization strategy, which involved a guanidium‐phosphate (Gu+/PO34‐) salt bridge to stabilize the electrostatic and hydrogen bond interactions between siRNA and polymers (Figure 7A). This design was reported to yield decreased *BACE1* expression, reduced A*β* level, and improved cognitive performance in the tested animals (Figure 7B–D). Some other natural materials have also been widely used to fabricate PNPs such as chitosan, dextran, and alginate.

Based on the charge, PNPs can be classified into cationic, anionic, and zwitterionic polymers which carry equal cationic and anionic groups in the same polymer chain.^[^ ^412^ ^]^ Specifically, the positively charged polymers, usually the amine‐containing polymers, are often used to encapsulate or adsorb negatively charged nucleic acids via electrostatic interaction, rendering them a useful tool for gene delivery for neural disorders.^[^ ^166^, ^413^ ^]^ A typical example is a work by Shi and co‐workers who developed a guanidinium and phenylboronic acid functionalized cationic PNPs to deliver negative siRNA for GBM treatment. Additional to electrostatic interactions, they also incorporated extra hydrogen and hydrophobic bond interactions to improve the physiological stability.^[^ ^414^ ^]^ Besides siRNA, this nanocarrier was recently demonstrated to co‐deliver anti‐miR‐21 and miR‐124 to modulate the RAS/P12K/PTEN/AKT pathways in GBM.^[^ ^415^ ^]^ It should be noted that the charge of some polymers could be altered under certain stimuli, such as pH, light, enzymes, temperature, and redox. This charge conversion, typically triggered by the cleavage of labile chemical bonds or the ionization/deionization of ionizable groups, provides the advantages of sustained delivery and triggered release of encapsulated genetic cargos. The nucleic acid delivery for CNS diseases can be facilitated by such a feature.

**Figure 8:**
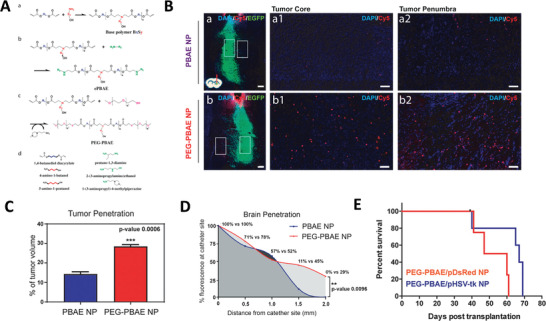
PEGylated NPs for gene delivery to the brain. A) Synthesis of polymers used for NP fabrication. a) Synthesis scheme of acrylate‐terminated PBAE base polymer, b) conventionally end‐capped ePBAEs, c) PEG–PBAEs, and d) monomers used in the synthesis of a library of PBAE‐based polymers. B) Confocal microscopy images of representative frozen sections from the brain injected with a) PBAE, b) PEG–PBAE NPs (scale bar = 200 µm). Higher magnification images of (a1/b1) tumor core regions and (a2/b2) tumor penumbra regions indicated by boxes respectively in (a) and (b) (scale bar = 50 µm). Blue: nuclei, Red: Cy5‐labeled NPs, Green: GBM1A cells. D) Tumor penetration of PEGylated and non‐PEGylated NPs. C) Brain penetration of PEGylated and non‐PEGylated NPs, analyzed as mean percent Cy5 fluorescence intensity in subdivided rectangular ROI in confocal images at 500 µm intervals beginning at the site of catheter implantation (*n* = 2, *p < 0.05). E) Kaplan–Meier survival plot between mice treated with intratumoral injection of either PEG–PBAE/pHSV‐tk or PEG–PBAE/pDsRed NPs in conjunction with systemic ganciclovir (*n* ≥ 4 per group). Reproduced with permission.^[^ ^418^ ^]^ Copyright 2020, American Chemical Society.

In addition to be directly used to form the core of NPs, polymers are also broadly used as surface modification materials for brain gene delivery. Among all, PEG of various molecular weight, surface density, and functional groups, are one of the most extensive polymers used (PEGylation). PEG on the surface not only provides NPs with a non‐fouling surface, which not only extends their blood circulation and leads to deeper brain parenchyma diffusion, but also decreases immunogenicity, surface aggregation, opsonization, and phagocytosis.^[^ ^416^, ^417^ ^]^ A typical example was carried out by Green et al., who conjugated PEG to poly (beta‐amino) ester (PBAE) NPs to co‐deliver plasmid DNA encoding suicide gene herpes simplex virus (HSV)‐derived enzyme thymidine kinase (HSVtk), and prodrug ganciclovir (**Figure** **8**A). Compared to PBAE NPs without PEG, PEG‐PBAE NPs showed improved gene transfection efficacies in the two tested GBM cells, including GBM1A cells (from 37% to 54%) and BTIC375 cells (from 66% to 82%). After convection‐enhanced delivery (CED)‐assisted administration, PEGylated NPs showed deeper brain penetration and increased the median survival rate of GBM mice from 53.5 to 67 days (Figure 8B–E).^[^ ^418^ ^]^ Other groups also had similar findings where PEG coating improved the brain penetration ability of tested NPs.^[^ ^419^, ^420^ ^]^ However, it should be noted that PEG‐modified NPs were locally administered into the brain by either direct diffusion or CED in these studies, bypassing the BBB, which is relatively invasive in the clinics. Indeed, PEGylated NPs have been reported to fail to cross the BBB.^[^ ^421^ ^]^ Apart from that, PEG may neutralize NP surface charge and decrease their efficiency to carry nucleic acids when electrostatic interactions are required.^[^ ^422^ ^]^ PEGylation may also reduce the binding affinity of other biological molecules on the NPs, such as targeting ligands, thus decreasing NP targeting ability. Therefore, extra attention should be paid by researchers before applying PEG coating.

**Figure 9:**
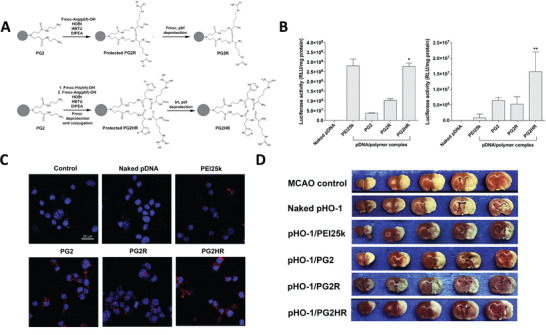
Gene delivery to the brain using dendrimers for ischemic stroke therapy. PAMAM generation 2 (PG2) conjugated with histidine and arginine (PG2HR) for delivery of plasmid DNA to the ischemic brain. A) Synthesis scheme of PG2 modified with only arginine (PG2R, top), and PG2 modified with both histidine and arginine (PG2HR, bottom). B) The transfection efficiency induced by the given treatments measured with a luciferase activity assay, in the absence of serum (left), and in the presence of serum (right). PEI25k was used as control. C) Confocal images of cellular uptake of plasmid DNA/polymer complexes(red). D) Infarct volume measured by TTC staining after delivery of plasmid DNA/polymer complexes in the ischemic brain. Plasmid DNA was designed to target HO‐1 gene (pHO‐1) encoding heme oxygenase‐1. MCAO‐reperfusion was performed as control. n = 7 in each group. Reproduced with permission.^[^ ^424^ ^]^ Copyright 2021, Elsevier B.V.

Dendrimers are also synthetic polymers intensively studied for CNS gene delivery. They are usually highly branched with 3D compact structures that consist of a central core and a corona.^[^ ^423^ ^]^ Different generations of dendrimers have been developed so far, among which poly(amido amine) (PAMAM) dendrimers are the most commonly explored. There are abundant internal functional groups in PAMAM, especially the primary and tertiary amine groups, which are protonated and provide a positively charged surface for negatively charged nucleic acids. Numerous attempts have been made to explore the application of dendrimers for CNS gene delivery. Like other polymer materials, dendrimers can be used to directly form the core of nanocarriers to accommodate the genetic cargos. For example, PAMAM (generation 2) was applied to form the core of a gene carrier for plasmid DNA delivery to treat ischemic stroke^[^ ^424^ ^]^ (**Figure** **9**A–D). In this study, histidine and arginine were conjugated to the primary amines of PAMAM to allow for the endosomal escape and thus improve the gene delivery efficiency. Many other brain‐specific moieties were used to conjugate to PAMAM to enhance the BBB penetration ability. For instance, Jin et al. first loaded siRNA against *LSINCT5*, a non‐coding RNA (ncRNA), to PAMAM NPs, which was then conjugated with CPPs tLyp‐1 to form the composite of tLypNP‐siRNA. To overcome the immunosuppressive microenvironment, an anti‐NKG2A monoclonal antibody (aNKG2A) was further conjugated onto tLypNP‐siRNA to form the composite of tLyp/aNKNP‐siRNA via a pH‐responsive linker. The formed nanocarriers efficiently delivered siRNA across the BBB and inhibited brain tumor growth as evidenced by in vitro and in vivo studies.^[^ ^13^ ^]^ Owing to the 3D structure and multi‐functionalization features, dendrimers are commonly used as a codelivery system for nucleic acids and other bioactive molecules.^[^ ^12^, ^425^ ^]^ For example, a study by Wei et al. showed that PAMAM NPs modified with RGD were used to deliver siRNA and anticancer drugs to brain cancer.^[^ ^12^ ^]^ In addition to forming the core of the carriers, dendrimers have also been used to modify other nanocarriers to enhance their solubility, bioavailability, and drug loading capacity. For example, graphene oxide NPs and selenium NPs became more stable and showed improved cellular selectivity after being modified with PAMAM. This strategy led to higher gene delivery efficiency and a better suppression of brain cancer cell growth.^[^ ^12^, ^426^ ^]^ PAMAM was also used to modify the electrodes to detect AD biomarker miRNA.^[^ ^427^ ^]^ However, the dense amine groups on the PAMAM surface, especially the higher generations, may cause potential cytotoxicity, which needs to be neutralized by surface modifications, or internal chemical composition.^[^ ^359^, ^428^, ^429^ ^]^ For example, AuNPs loading not only decreased the cytotoxicity of PAMAM material, but also improved their siRNA delivery efficiency for GBM treatment.^[^ ^359^, ^430^ ^]^ Nevertheless, for better application in brain gene delivery, more investigations need to be done to address other challenges underlying dendrimers, such as complicated and high‐cost synthesis process, and unpredictable drug release.

Amphiphilic block copolymers (BCPs) featuring a narrow distribution of molecular weight and compartmentalized segments are another polymeric material widely explored in CNS gene delivery. They can self‐assemble into highly ordered multimolecular architectures, including micelles and hollow vesicles (usually spheres, termed as polymersomes). BCPs usually consist of a hydrophobic core and hydrophilic internal and external coronas.^[^ ^431^, ^432^ ^]^ Their high solubility and low toxicity are attractive attributes for gene delivery.^[^ ^433^, ^434^ ^]^ For example, micelles formed by the deoxycholic acid‐conjugated polyethyleneimine (DA‐PEI) have been reported to efficiently deliver heme oxygenase‐1 (*HO‐1*) gene‐targeting plasmid DNA and miR21‐targeting AON for stroke and GBM treatment, respectively.^[^ ^435^, ^436^ ^]^ Further surface modifications allow BCP assemblies to enhance the cell penetration and BBB permeability.^[^ ^437^, ^438^ ^]^ For instance, Kanazawa and co‐workers loaded siRNA against TNF‐*α* into a composite of PEG‐PCL‐Tat, which was formed by conjugating the PEG‐PCL micelles with a targeting peptide Tat. siRNA delivered by the established nanocarriers through intranasal administration lowered tTNF‐*α* production and neurological score of a rat model of cerebral stroke.^[^ ^439^ ^]^ Another study carried out by Shi et al. showed that angiopep‐2 (A2)‐modified PEG‐PEI‐PCL micelles can deliver siRNA and drugs to brain tumor both in vitro and in vivo.^[^ ^438^ ^]^ Additionally, the polymersomes have been also used to the codelivery of nucleic acids and other smaller NPs to achieve multiple functions. For example, Lu et al. developed a polymersome to codeliver superparamagnetic iron oxide NPs and siRNA into the NSC, which were subsequently transplanted into the striatum ipsilateral of the ischemic rat brain. Along with the MRI guidance, such an approach achieved silencing of the target gene *NgR* encoding Nogo‐66 receptors and improved the recovery of neural function in the tested animals.^[^ ^381^ ^]^

### 5.3 Carbon Nanotubes

Carbon nanotubes (CNTs) are nanotubes with cylindrical shapes consisting of a hexagonal arrangement of sp^2^ hybridized carbon atoms (also called graphene).^[^ ^440^ ^]^ Based on the number of layers, CNTs can be classified into single‐walled CNTs (SWCNTs), which have single‐rolled sheets of graphene, and multi‐walled CNTs (MWCNTs), which have rolled‐up structures of multiple concentric graphene sheets.^[^ ^441^ ^]^ Due to the innate hydrophobic properties, CNTs are generally difficult to disperse in the aqueous phase. Surface modification is essential not only to improve their solubility and biocompatibility but also to improve their targeting ability. SWCNT usually has the size of 0.4–2 nm with a length up to a few µm, while the size of MWCNT can range from 2–100 nm with a length up to several tens of µm.^[^ ^442^, ^443^ ^]^ Owing to their small size, high surface area to volume ratio, and high ability to contain bioactive molecules, CNTs show great potential for the delivery of gene therapy for CNS diseases.

CNTs themselves can trigger therapeutic effects towards different CNS diseases. For example, SWCNT treatments sufficiently suppressed autophagic and lysosomal defects in primary glial from a mouse model of AD.^[^ ^444^ ^]^ An in vivo rat stroke model also demonstrated amine‐functionalized SWCNTs as neuroprotective agents.^[^ ^445^ ^]^ CNTs have a high capacity to absorb light in the near‐infrared (NIR), which has been used for thermal therapy for GBM treatment. Following NIR exposure, CNTs can generate the substantial amounts of heat and thus induce hyperthermia, which has been shown to inhibit GBM progression in vitro and in vivo.^[^ ^446^ ^]^ The NIR fluorescence of CNTs also renders them an attractive nanosensor for brain imaging.^[^ ^447^ ^]^ Kruss et al. reported a single‐stranded DNA‐ and RNA wrapped in SWCNTs can act as conformational switches to modulate the SWCNT fluorescence, which could be used for neurotransmitter detection.^[^ ^448^ ^]^

CNTs can efficiently penetrate into cells and enter the nucleus, therefore allowing for both cellular and nuclear delivery of genetic materials.^[^ ^449^ ^]^ Generally, MWCNTs are considered to be able to accommodate a larger number of therapeutic materials than SWCNTs in their inside walls owing to their larger diameters. Genetic materials can be attached CNTs covalently at either the end or the sidewalls. Such functionalizations are usually achieved by acidic treatments (e.g., HNO_3_) or by cycloaddition reactions at the sp^2^ carbon skeleton of CNTs to yield carboxylic acidic groups on the CNTs first, which can be further conjugated with various molecules, such as peptides, proteins, antibodies, and genetic materials.^[^ ^450^ ^]^ Owing to the large aromatic surface, CNTs can also non‐covalently entrap molecules on the tubular surface (exohedral functionalization) or within the inner area (endohedral functionalization) via *π*‐*π* stacking and van der Waals interactions.^[^ ^440^ ^]^ It has been reported that siRNA delivered by ammonium‐functionalized MWNTs effectively suppressed the expression of the target gene that encoded caspase‐3, which significantly reduced neurodegeneration and led to functional improvement in a rat model of ischemic stroke.^[^ ^451^ ^]^ However, it is noted that though the application of CNTs in gene therapy delivery is rapidly expanding for other disease studies, their application for gene therapy delivery to the brain is still at its early stage. A study by Eldridge et al. showed that MWCNTs (8–15 nm) coated with phospholipid‐PEG allowed for sufficient diffusion into brain extracellular matrix (ECM)‐mimicking phantoms.^[^ ^452^ ^]^ But more studies are needed to further evidence their ability to cross the BBB, and demonstrate the toxicity file of CNTs in the brain.

### 5.4 Exosomes

Exosomes are natural extracellular nanovesicles (<150 nm) with hydrophilic bilayers secreted by various cell types. A number of CNS cells and other cells have been found to possess the ability to release exosomes, such as HEK‐293, dendritic cells, MSCs, and glioma cells. Exosome‐based gene delivery platform provides many unique biological properties low immunogenicity, high biocompatibility and versatility, and low aggregation potential.^[^ ^453^, ^454^ ^]^ Different from other synthetic nanocarriers, exosomes are involved in various physiological and pathological processes in the immune system and nervous system.^[^ ^455^ ^]^ They are one of the intercellular communicators by transporting regulatory and functional molecules, proteins, and nucleic acids.^[^ ^456^ ^]^ Owing to these natural features, exosomes are rich in parental membrane transportation and fusion proteins (e.g., GTPase, annexin) and specific surface markers (e.g., CD9, CD63, CD81), which could potentially be recognized by the recipient cells such as neuronal cells.^[^ ^457^ ^]^ Moreover, exosomes originating from certain cell types, for example, adipose‐derived MSCs, bone marrow MSCs, and glioma cells, display the natural ability to cross the BBB and other biological barriers. These render exosomes as a useful tool for the gene delivery to the brain as exemplified in in stroke, AD, and PD animal models.^[^ ^458^, ^459^ ^]^

**Figure 10:**
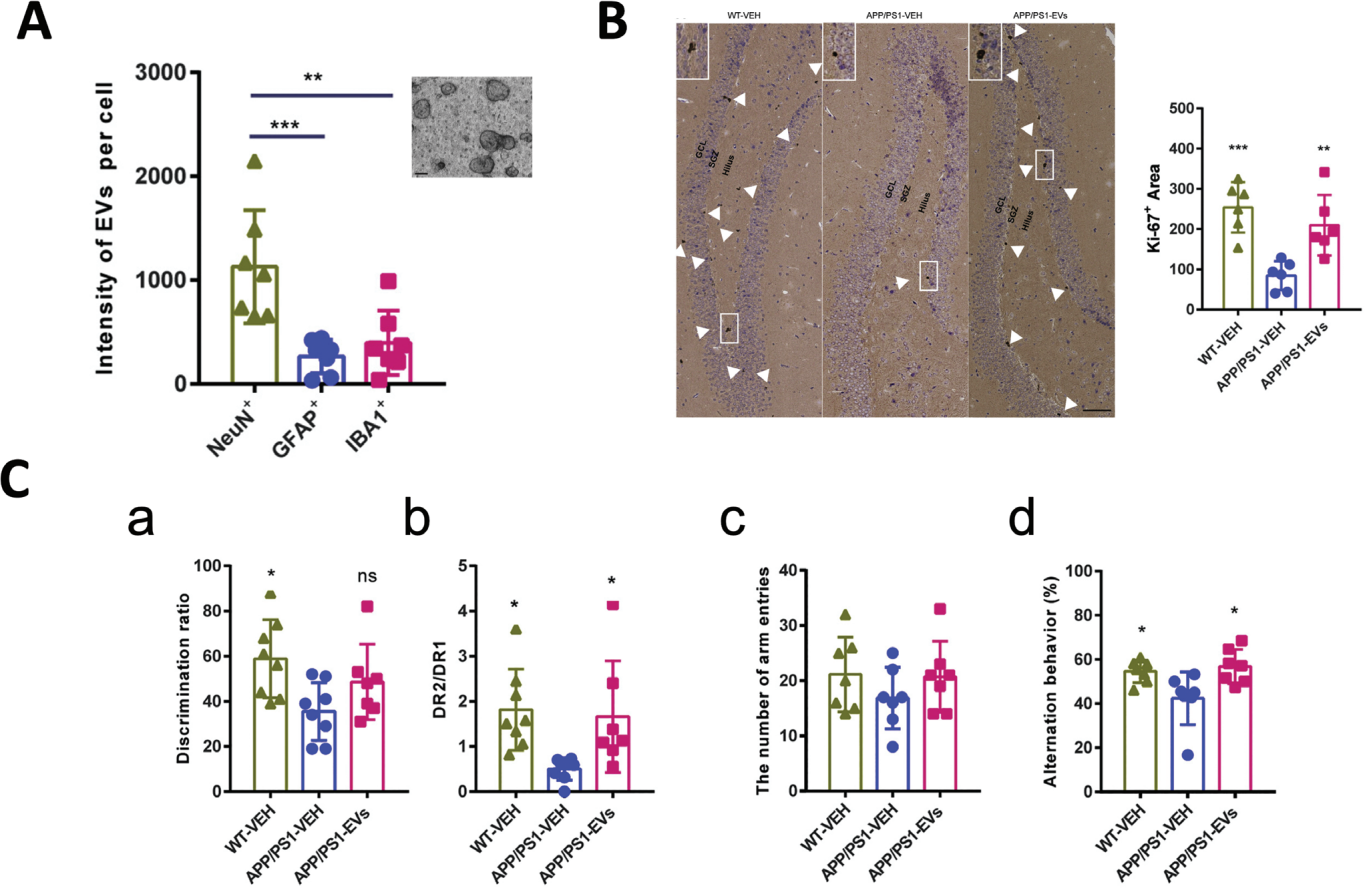
Brain gene delivery using exosomes for AD. Exosomes derived from MSCs (MSCs) for delivery for AD. A) Size distribution of exosomes measured by DLS. B) TEM images of exosomes. scale bar = 100 nm. C) The brain distribution of exosomes in the AD brain after 1 h intranasal administration in APP/PS1 mice. They were mainly found in the neurons (NeuN) and much less in astrocytes (GFAP) and microglia (IBA‐1). (B) Exosomes increased Ki‐67 positive cells in the DG region of hippocampus in APP/PS1 mice, indicating their neuroprotection efficacy. Left: Immunohistochemical staining for Ki‐67. right: quantification of Ki67‐67 positive cells. (C) Exosomes rescued memory deficits in APP/PS1 mice by showing improved a) DR in the novel object recognition test; b) the ratio of DR in the second day (DR2) and the first day (DR1) in the novel object recognition test; c) the number of arm entries and d) alternation behavior in Y‐maze test. Data represent mean ± SD, n = 7–8 per group, *p<0.05, **p<0.01, ***p< 0.001, compared with the vehicle treated APP/PS1 group. Reproduced with permission.^[^ ^462^ ^]^ Copyright 1969, Elsevier.

Exosomes have become one of the most actively exploited nanocarriers in the delivery of nucleic acids to the brain. A milestone work done by Matthew and colleagues in 2011 successfully engineered HEK‐293‐derived exosomes and applied them to deliver siRNA targeting the disease risk gene *BACE1* to the brain in a mouse AD model, harvesting the reduced gene expression at mRNA and protein levels both in vitro and in vivo.^[^ ^460^ ^]^ Following this study, Didiot et al. reported that exosomes were loaded siRNA against *HTT* and delivered to mouse striatum by stereotaxic injection, inducing *HTT* mRNA knockdown by more than 35% in vivo.^[^ ^180^ ^]^ However, both works did not report the actual therapeutic efficacies of the tested exosomes in the treated animal. Such a gap is recently filled by Yavar et al.^[^ ^461^ ^]^ In this study, they harvested the exosomes from the transfected rat bone marrow MSCs and HEK‐293 cells, which carried miR‐29a targeting *BACE1* and miR‐29b targeting *BIM* (Bcl‐2 interacting mediator of cell death). These exosomes (50–171 nm) were subsequently injected into the *conru ammonis* of AD rats, which improved the spatial learning and memory ability of animals though only to a limited extent. Ma et al. also reported that the exosomes derived from adipose MSCs by ultracentrifuge were able to quickly enter the brain following intranasal administration without any modification and additional cargo loading. They found the exosomes mainly accumulated in neurons within CNS, which increased newborn neurons and promoted neurogenesis in APP/PS1 transgenic AD mice (**Figure** **10**A–C).^[^ ^462^ ^]^ Meanwhile, several independent groups attempted to engineer exosomes with Syn‐targeted genetic cargos, such as shRNA minicircles and aptamer for PD gene therapy.^[^ ^47^, ^463^ ^]^ In Ren's study, the exosomes harvested from HEK‐293 cells were modified with neuron‐specific rabies viral glycoprotein (RVG) peptides and then loaded with DNA aptamer that targeted α‐Syn. After intraperitoneal injection, these exosomes decreased α‐Syn expression and aggregation, reduced loss of dopaminergic neurons, and reduced the neuropathological deficits of PD mice.^[^ ^463^ ^]^ Exosomes were also engineered with miR‐21‐targeted AON or miRNA, which decreased the tumor size and prolonged the survival in xenograft GBM mice models.^[^ ^168^, ^464^ ^]^ Notably, Yang et al. reported that the circular RNA‐contained exosomes improved the functional recovery in both mice and monkey models of stroke.^[^ ^203^ ^]^ Currently, two clinical trials are being carried out to study the naïve exosomes (NCT04388982 for AD) or gene‐contained exosomes (NCT03384433 for stroke) for brain disease treatment although there has been no outcome reported so far.

**Figure 11:**
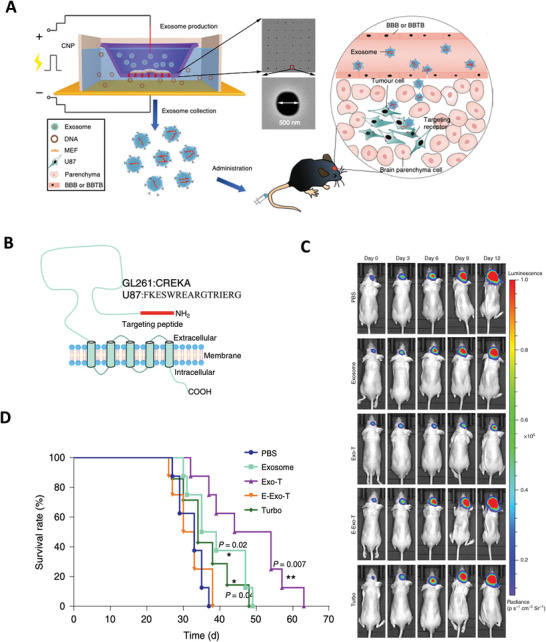
CNP for large‐scale production of exosomes for mRNA delivery in GBM. A) Schematic of CNP by using transient electrical pulses. The harvested exosomes contain transcribed mRNA and can cross the blood‐brain‐barrier (BBB) or blood‐brain‐tumor barrier (BBTB). B) Schematic of cloning GBM‐targeting peptides into the N terminus of CD47 on the surface of exosomes. C) Survival curve of mice with orthotopic GBM received treatments of exosomes (exosomes), Exo‐T containing *PTEN* mRNA (Exo‐T), empty Exo‐T (E‐Exo‐T), TurboFect NPs (Turbo), or PBS via tail‐vein injection. n = 8 mice per group. Reproduced with permission.^[^ ^465^ ^]^ Copyright 2019, Springer Nature Limited.

Exosomes membrane modification can improve their targeting efficiency. Although natural exosomes possess the cell‐derived moieties, the exosomes commonly studied largely originated from non‐neuronal cells such as dendritic cells or kidney cells, which may lack the ability to target brain cells. Surface engineering could enhance their brain targeting capability, reduce opsonization and sequestration, and control cargo release.^[^ ^453^ ^]^ A typical approach is to fuse the exosomal outer membrane with different neuron‐specific peptides such as RVG and T7, which could be achieved by either transfecting the exosome‐produced cells with pRVG‐lamp2b/pT7‐Lamp2b,^[^ ^168^, ^203^ ^]^ or by post decorating exosomes with targeted peptides.^[^ ^465^ ^]^ The targeted exosomes harvested from both approaches have been applied in gene therapy for PD, AD, stroke, and GBM.^[^ ^168^, ^203^, ^461^, ^463^ ^]^ In 2020, Yang et al. reported a remarkable design termed as cellular nanoporation (CNP), which strikingly stimulates the production of exosomes with enriched therapeutic mRNA.^[^ ^465^ ^]^ In this design, mouse embryonic fibroblast (MEF) cells were transfected with plasmid DNA by using an in‐house biochip and subsequently stimulated with a transient electrical stimulus. Compared with electroporation and other traditional stress‐induced exosome methods, CNP was reported to increase exosome production over 50‐folds and exosomal mRNA transcripts over 10^3^‐folds. The authors further cloned glioma‐targeting peptides into a transmembrane protein (CD47) on the surface of exosomes, including CDX peptides for U87 cells and CREKA peptides for GL261 cells. The harvested exosomes sufficiently delivered mRNA against *PTEN* to the brains in two different orthotopic GBM mouse models, which showed suppressed tumor growth and increased survival rate (**Figure** **11**A–D). The high exosome production by CNP was also shown in dendritic cells, MSCs, and HEK‐293 cells. It may provide an instructive method to break the limitation of cell types used to produce exosomes that the field is currently facing.

**Figure 12:**
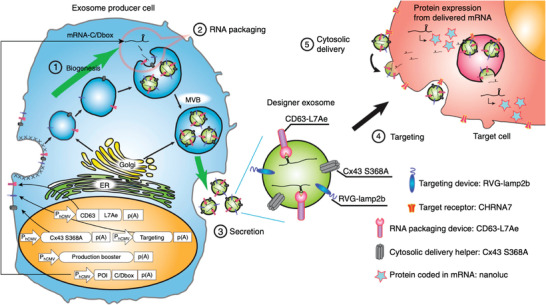
Schematic of EXOtic devices for mRNA delivery. Exosomes containing the RNA packaging device (CD63‐L7Ae), targeting module (RVG‐Lamp2b), cytosolic delivery helper (Cx43 S368A), and mRNA (e.g., nluc‐C/Dbox), were produced from exosome producer cells by means of an exosome production booster. Reproduced under the terms of Creative Commons Attribution 4.0 International License.^[^ ^293^ ^]^ Copyright 2018, The Author(s), published by Spring Nature Group.

Recent breakthroughs in technology in cellular therapy and synthetic biology also boosted the development of exosome‐based gene delivery in brain disease. In work by Kojima et al.,^[^ ^293^ ^]^ the authors reprogrammed human‐derived HEK‐293 cells with the discovered exosome production‐booster genes (e.g., *STEAP3*, *SDC4*, and *NadB*), cytosolic delivery helper (Cx43 S368A), RNA packaging device (CD63‐L7Ae), genes encoding targeting molecules (RVG), and mRNA (**Figure** **12**) to produce exosomes. Such a design was reported to increase the exosome production by over 50‐folds. They achieved the desirable preclinical results in in vitro and in vivo PD models, including the enhanced delivery efficiency of adequate mRNA, reduced neurotoxicity, and neuroinflammation. This synthetic exosome‐based gene delivery strategy offers a robust and feasible approach to controlling the cell‐to‐cell communication at will without the need to concentrate exosomes.

Nevertheless, the research on exosomes in brain disease research is still in its infancy. The application of exosomes needs to overcome several key challenges. First, the current understanding of the specific roles that exosomes are involved in the biological process in the CNS and other tissues remains preliminary. Studies show that some exosomes could be of importance in neuroprotection and neurodegeneration, whilst some exosomes mediate the neurodegenerative diseases, and some are associated with the tumorigenesis, metastasis, and chemotherapy resistance in brain tumors as they carry disease proteins/nucleic acids.^[^ ^455^, ^466^, ^467^ ^]^ Second, it is essential to increase the production efficiency and reproducibility of desired exosomes, as well as improve the cargo encapsulation efficiency and transfection efficiency. The current isolation, purification, and characterization protocols of exosomes have not been well defined, which could be reflected by the non‐standard approaches to acquiring/analyzing the data and nomenclature. To obtain exosomes of desirable structural and compositional integrity, a number of factors should be taken into consideration, such as size, density, cell origins, and biochemical composition. Indeed, it is important to balance the production efficiency, transfection efficiency, and immunogenicity. For example, although tumor cells such as GBM cells have shown an ability to produce a high level of exosomes,^[^ ^180^ ^]^ the application of tumor cell‐derived exosomes still remains controversial as multiple researchers have demonstrated the pathogenetic role exosomes are involved in.^[^ ^468^ ^]^ Nevertheless, it is believed that further innovations in nanotechnology, synthetic biology, and surface engineering will push forward the development of exosome‐based gene delivery for the treatment of brain diseases.

### 5.5 Lipid‐based NPs

**Figure 13:**
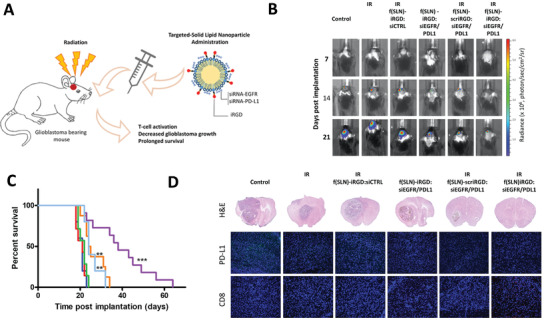
Brain gene delivery using liposomes for GBM. A) Schematic of solid lipid NPs (SLN) with targeting peptide iRGD for delivery of siRNA against EGFR and PD‐L1 for GBM. A low dose of radiation was given to mice with an orthotopic GBM alongside the retro‐orbital administration of treatments. B) Florescence images of tumor growth in mice bearing GL261‐Fluc tumors. Mice were irradiated (or not as a control), and retro‐orbitally injected with either f(SLN)‐iRGD:siCTRL, f(SLN)‐iRGC:siEGFR/PLD1, or f(SLN)‐scriRGD:siEGFR/PDL1. The treatments were given at days 7, 14, and 21 after tumor cell inoculation. C) Kaplan–Meier survival curves of GBM mice received different treatments. n = 5–12 mice per group. D) H&E staining (top), immunohistochemical staining of PD‐L1 (middle), and CD8 (bottom) of brain sections in GBM mice receiving various treatments (DAPI, blue; PD‐L1, green; and CD8, red). The combination of radiation and nanocomplexes decreased PD‐L1 expression while increasing recruitment of CD8^+^ T‐cells. Reproduced with permission.^[^ ^158^ ^]^ Copyright 2019, American Chemical Society.

Lipid‐based NPs (LNPs) are vesicles containing an aqueous inner core with phospholipid bilayers. Typically, they are made from biodegradable and biocompatible materials that contain polar head groups (hydrophilic) and nonpolar tails (hydrophobic), such as cholesterol, phospholipids, and fatty acids.^[^ ^469^ ^]^ The bilayer components enable LNPs to encapsulate both hydrophobic and hydrophilic bioactive molecules, including nucleic acids, into the lipid bilayer or aqueous cores. Cationic lipids, such as 1,2‐di‐*O*‐octadecenyl‐3‐trimethylammomium‐propane (DOTMA) lipid and 1,2‐dioleoyl‐3‐trimethylammonium‐propane (DOTAP), and zwitterionic lipids, such as 1,2‐dioleoyl‐sn‐glycero‐3‐phosphoethanolamine (DOPE), have been used alone or in combination with other materials for brain gene delivery.^[^ ^470^, ^471^ ^]^ These charged LNPs usually consist of a helper lipid to stabilize the formulation, and a PEGylated lipid to reduce the opsonizations and reticuloendothelial clearance.^[^ ^472^, ^473^ ^]^ It should be noted that the ratio of various components can significantly affect the corresponding gene delivery efficiency.^[^ ^474^ ^]^ LNPs are emerging as promising nanocarriers of genetic molecules, such as siRNA,^[^ ^475^, ^476^ ^]^ miRNA,^[^ ^470^ ^]^ and plasmid DNA^[^ ^138^ ^]^ for brain gene delivery in the past decades. For example, Lin et al. applied the liposomes to deliver *GDNF*/*BDNF* plasmid gene for PD treatment,^[^ ^138^ ^]^ while Rassu and colleagues used lipid to deliver siRNA against *BACE1* to an AD model.^[^ ^475^ ^]^ A study carried out by Gulsah and colleagues applied iRGD‐modified solid lipid NPs to deliver siRNA against *EGFR* and programmed cell death ligand‐1 (PD‐L1) along with a low‐dose radiation. They observed that this combination of gene therapy, immunotherapy, and radiation therapy sufficiently decreased the expression of targeted genes and prolonged the survival of the GBM‐bearing mice (**Figure** **13**A–D).^[^ ^158^ ^]^ In 2020, LNPs were approved by the FDA for mRNA delivery for COVID‐19 vaccines, which highlights their huge potential in the gene therapy field.^[^ ^477^ ^]^

**Figure 14:**
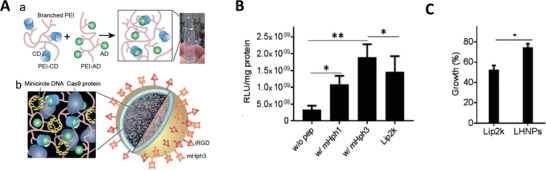
Brain gene delivery using liposomes for GBM. A) Schematics of liposome‐templated hydrogel NPs (LHNPs) where the core is made from DOTAP liposomes and the shell is a PEI hydrogel (a). The surface is modified with targeting peptides mHph3 and internalizing RGD (iRGD) by conjugation (b). In this study, lipofectamine 2000 (Lip2k) was used as benchmark. B) Gene delivery efficiency of pGL4.13‐loaded, PEI hydrogel‐core DOTAP liposomes and Lipofectamine 2000 (Lip2k) on U87 cells as measured with Luciferase signal. The treatments were given for 72 h. C) Cytotoxicity of LHNPs and Lip2k on U87 cells when 60 ng of DNA was delivered. Reproduced with permission.^[^ ^32^ ^]^ Copyright 2017, WILEY‐VCH.

Some liposome formulations such as lipofectamine have already been commercialized for decades for in vitro and in vivo delivery of nucleic acids. But the attractive transfection efficacy of lipofectamine and other cationic liposomes is compromised by the findings that they could cause cytotoxicity and immunogenicity.^[^ ^478^ ^]^ Numerous studies are ongoing to develop the alternative or optimized LNPs, in which lipofectamine products are often used as benchmarks.^[^ ^470^ ^]^ For example, Chen et al. fabricated the PEI‐based hydrogel within the cationic liposomes to deliver sgRNA‐Cas9 for GBM, which showed a better efficiency than lipofectamine 2000 (**Figure** **14**A–C).^[^ ^32^ ^]^ In this study, instead of directly delivering the Cas9 gene, the authors encapsulated Cas9 protein together with the anionic minicircle sgRNA into liposomes. Being further decorated with targeting ligand iRGD and cell penetration peptide mHpH3, the as‐designed nanocarrier system efficiently inhibited the growth of two GBM cell lines and reduced the tumor volume in vivo.

**Figure 15:**
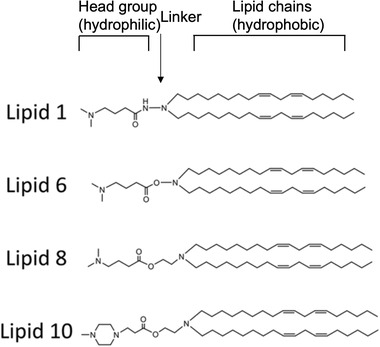
Ionizable lipid for nucleic acid delivery to the brain. Chemical structures of the selected lipids, which typically contains a head group (hydrophilic), lipid chain (hydrophobic), and a linker. Reproduced with permission.^[^ ^311^ ^]^ Copyright 2020, The Authors, some rights reserved, exclusive licensee AAAS. Distributed under a Creative Commons Attribution NonCoomercial License 4.0 (CC‐BY‐NC).

Studies have also been carried out to develop ionizable lipids to increase gene delivery efficiency. Typically, an ionizable amino lipid contains three major compositions: a hydrophilic amine head, a hydrophobic lipid chain, and a linker connecting two parts (**Figure** **15**).^[^ ^311^ ^]^ At physiological pH, these lipids possess a neutral charge, while at low pH environment they are protonated and tend to be cationic.^[^ ^479^ ^]^ Such a pH‐sensitive feature will benefit nucleic acid delivery in vivo, because neutral lipids will have less interactions with anionic membrane of blood cells, and thus be more biocompatible. While trapped in the acidic endo‐lysosomal compartments, ionizable lipids are protonated and hence become positively charged, which may destabilize the membrane and thus escape from endosome hydrolysis.^[^ ^477^, ^479^ ^]^ In 2018, Patisiran, a siRNA‐based drug that contains the amino lipid DLin‐MC3‐DMA (MC3) has obtained FDA‐approval for polyneuropathy treatment. Following this, Rosenblum et al. intracerebrally administered the DLin‐MC3‐based ionizable lipids to deliver CRISPR‐Cas9 mRNA and sgRNA, which yielded high gene editing efficiency (>70%) and tumor growth suppression (50%) in mice with orthotopic GBM.^[^ ^183^ ^]^

## 6 Outlook and Conclusions

In this review, we focus on the current state of the art in the field of nanomedicines‐based science, and its applications in delivering gene therapies for CNS diseases, focusing on the underlying design rationale. Despite the promising preclinical success, the current progress on the nanomedicine‐assisted gene delivery to CNS has rarely been seen in human studies so far. **Table** **2** represents the overview and recent trends in clinical trials applying non‐viral nanomedicine for gene delivery in brain diseases. As shown in the table, only a relatively limited number of preclinical studies have converted into clinical studies, with GBM and AD accounting for most of them. Several major challenges still have to be addressed to translate these nanomedicines to clinical practice:

– The ability to penetrate the BBB and other biological barriers, such as blood‐brain tumor barrier (BBTB) and blood‐cerebro‐spinal‐fluid barrier (BCSFB), remains to be the main challenge for most of the nanocarriers.^[^ ^480^, ^481^ ^]^ To overcome the hurdle of the BBB and other biological barriers, several strategies have been explored in various studies: a) Functionalize the nanocarriers with the brain‐targeting moieties as introduced throughout this review, including a range of proteins (e.g., transferrin, insulin, RGV, LDL‐receptor‐related protein, SLC protein), CBSA, polymers, peptides, and nucleic acids. These biofunctionalizations allow for the active transportation of nanomedicines through the transcytosis, such as receptor‐mediated endocytosis (e.g., transferrin receptors, insulin receptors, scavenger receptors, glutamate receptors). b) Apply the cell‐mediated gene delivery, and increase the homing ability of certain stem cells, such as MSCs and NSCs.^[^ ^464^, ^482^, ^483^ ^]^ c) Disrupt the BBB by the physical methods for a short time while delivering the nanomedicine, such as MRI‐guided FUS, and radiation.^[^ ^381^, ^484^ ^]^ d) Influence the efflux systems at the BBB, which are typically built up by the efflux transporters, such as ATP‐binding cassette (ABC) family (e.g., MDR1 proteins, also known as P‐glycoprotein), solute carrier (SLC) family and organic anion transporters (OAT).^[^ ^485^, ^487^ ^]^
– Off‐target accumulation is still a considerable obstacle for these brain nanomedicines, which leads to the potential requirement of the high or repeating dosing, thus increasing their risk of toxicity and side effects. Therefore, it is essential to increase their brain perfusion and neuronal targeting ability, while avoiding the fast renal and reticuloendothelial clearances. Researchers have attempted to address this issue by various approaches, such as involving the targeting ligands into the delivery system, and/or controlling the release of encapsulated genetic materials. However, despite CNS diseases sharing some common phenomena that could be targeted, there will not be any treat‐for‐all nanomedicines that can provide effective treatment for all CNS diseases, but rather a preferred therapy for a particular target and disease. For instance, a CRISPR‐Cas9 nanocomplex has been shown to be more effective in focal neurodegenerative disorders like PD, than others with widespread disorders in the neuronal circuit, such as AD.^[^ ^95^ ^]^ Therefore, it is of importance to rationally select the suitable genetic modulators as well as the type of nanocarriers at the early designing stage.
– The disease genetic networks, molecular mechanisms, and biological progression remain largely unexplored for most CNS diseases. Though various disease‐implicated genes have been discovered as mentioned above, a further understanding of their biological functions is needed. In addition, given that many of the current discoveries of the mechanisms and biological pathways for disease progression are either not understood or controversial, massive efforts are still needed to deepen our knowledge in this field. It should be noted that many animal models used in preclinical studies can hardly reflect all the phenotypic characteristics of their corresponding CNS diseases, which increases the challenges to evaluate the therapeutic efficacy of the gene therapies. Therefore, it is essential to establish more clinically relevant CNS disease models, which will in turn benefit the translation of preclinical discoveries to the clinic setting.
– Degradation products of the nanomedicines and their bioeffects on normal physiological behaviors of cells/tissues remain poorly understood. Generally, most of the studies claim their nanomaterials had limited side effects on the non‐targeted cell and tissues during the application of CNS gene delivery at optimum dose and/or concentrations. Nevertheless, attention should be paid to investigating the concentration‐dependent toxicity of nanomaterials in both preclinical and clinical studies. Another challenge relates to the concern that gene editing is irreversible, and in most cases, uncontrollable. However rare this may be, researchers should pay extra attention to avoid genomic alterations while applying the various gene therapies.
– Large‐scale manufacturing of nanomedicines remains a big challenge. Successful nanomedicines require a reliable platform that could reproducibly produce uniform nanomedicines meeting the material demands. Ideally, they should have the desired physicochemical properties (e.g., size, charge, shape), sound safety and efficacy, controlled pharmacokinetic and biodistribution features, and scalable for large production. Last but not least, the cost of manufacturing and commercialization should also be taken into consideration. In this regard, a 5’R's framework, including “right target”, “right tissue”, “right safety”, “right patients”, and “right commercial potential”, has been brought up to accelerate the success of the new drug translation to the clinic.^[^ ^488^ ^]^ Using a decision‐making strategy like the 5Rs may benefit drug discovery and development, including delivering the target genetic materials to the brain for medical applications.

**Table 2:** Non‐viral nanovectors for CNS gene delivery that have entered clinical trials

Non‐viral Vector	Genetic cargo carried	Targeted gene	Investigated indication	Status	Milestones	Originator	Identifier (Phase)
Gold NPs	Spherical Nucleic Acid	Bcl2L12	Recurrent GBM, or GBM undergoing surgery	Completed	Start date: Nov 13, 2017 Last update date: Oct 27, 2020	Northwestern University	NCT 03020017 (Early Phase I)
Cationic Liposome	cDNA	P53	Recurrent GBM	Terminated	Start date: Jan 16, 2015 Last update date: Mar 3, 2021	SynerGene Therapeutics, Inc.	NCT02340156 (Phase II)
Dendritic cells	mRNA (electroporated)	WT1	GBM and other solid tumors	Unknown	Start date: Feb 8, 2011 Last update date: Jul 12, 2013	University Hospital, Antwerp	NCT0191420 (Phase I/II)
Autologous T‐Cells	‐	T‐cell receptor gene	GBM	Recruiting	Start date: Sep 25, 2019 Last update date: Mar 24, 2022	National Cancer Institute (NCI)	NCT04102436 (Phase II)
Unknown	Plasmids DNA	WT1 antigen, PSMA antigen, hTERT genes	Newly‐diagnosed GBM	Active, not recruiting	Start date: Apr 9, 2018 Last update date: Mar 3, 2022	Inovio Pharmaceuticals	NCT03491683 (Phase I/II)
Neural stem cells	unknown	E.Coli Cytosine deaminase	Recurrent GBM	completed	Start date: Jul 30, 2010 Last update date: Nov 9, 2017	City of Hope Medical Center	NCT01172964 (Phase I)
Mesenchymal stem cells	Unknown	Suicide gene cytosine deaminase (CD)	Recurrent GBM	Enrolling by invitation	Start date: Dec 8, 2020 Last update date: Jan 11, 2022	CHA University, Ajou University School of Medicine	NCT04657315 (Phase I)
ARPE‐19 cells	NGF gene	NGF gene	AD	Unknown	Start date: Jul 16, 2010 Last update date: Jul 16, 2010	NsGene A/S	NCT01163825 (Phase I)

To sum up, recent progress in relation to gene therapy‐based nanomedicines has led to a massive increase in applications to CNS diseases. Many of them have shown an ability to target the disease‐implicating genes and achieve to yield an efficient therapeutic efficacy in the preclinical studies. Though clinical translation and commercialization are limited so far due to various obstacles, there has been remarkable progress in terms of CNS disease treatments. We believe that a multidisciplinary approach combining the effort of biology, material, chemistry, and pharmaceutics would no doubt achieve breakthrough development and translate the promise of gene therapy into medical practice in the future.

## Conflict of Interest

The authors declare no conflict of interest.
